# FORGEdb: a tool for identifying candidate functional variants and uncovering target genes and mechanisms for complex diseases

**DOI:** 10.1186/s13059-023-03126-1

**Published:** 2024-01-02

**Authors:** Charles E. Breeze, Eric Haugen, María Gutierrez-Arcelus, Xiaozheng Yao, Andrew Teschendorff, Stephan Beck, Ian Dunham, John Stamatoyannopoulos, Nora Franceschini, Mitchell J. Machiela, Sonja I. Berndt

**Affiliations:** 1 Division of Cancer Epidemiology and Genetics, National Cancer Institute, National Institutes of Health, Bethesda, MD 20892 USA; 2 Altius Institute for Biomedical Sciences, 2211 Elliott Avenue 98121, Seattle, USA; 3 UCL Cancer Institute, University College London, 72 Huntley Street, London, WC1E 6BT UK; 4 Division of Immunology, Department of Pediatrics, Boston Children’s Hospital, Harvard Medical School, Boston, MA USA; 5 Broad Institute of MIT and Harvard, Cambridge, MA USA; 6 CAS Key Lab of Computational Biology, Shanghai Institute for Biological Sciences, CAS-MPG Partner Institute for Computational Biology, Chinese Academy of Sciences, 320 Yue Yang Road, Shanghai, 200031 China; 7 European Molecular Biology Laboratory, European Bioinformatics Institute (EMBL-EBI), Wellcome Genome Campus, Hinxton, Cambridge, CB10 1SD UK; 8 Department of Epidemiology, University of North Carolina, Chapel Hill, NC USA

**Keywords:** Gene regulation, Functional annotation, Variant scoring, Regulatory elements, Genome-wide association study (GWAS), Expression quantitative trait locus (eQTL), Massively parallel reporter assay (MPRA), Activity-by-contact (ABC), DNase-seq, Transcription factor (TF), CRISPR (clustered regularly interspaced short palindromic repeats), Single guide RNA (sgRNA)

## Abstract

The majority of disease-associated variants identified through genome-wide association studies are located outside of protein-coding regions. Prioritizing candidate regulatory variants and gene targets to identify potential biological mechanisms for further functional experiments can be challenging. To address this challenge, we developed FORGEdb (https://forgedb.cancer.gov/; https://forge2.altiusinstitute.org/files/forgedb.html; and https://doi.org/10.5281/zenodo.10067458), a standalone and web-based tool that integrates multiple datasets, delivering information on associated regulatory elements, transcription factor binding sites, and target genes for over 37 million variants. FORGEdb scores provide researchers with a quantitative assessment of the relative importance of each variant for targeted functional experiments.

**Supplementary Information:**

The online version contains supplementary material available at 10.1186/s13059-023-03126-1.

## Background

Genome-wide association studies (GWAS) have been remarkably successful in identifying genetic loci associated with many different diseases and traits [1]. As of the end of 2022, the GWAS catalog comprised > 232,000 distinct variants associated with > 3000 diseases and traits [2]. Many loci identified from GWAS are intergenic, locating to non-protein-coding regions of the genome [3]. Although the functional mechanisms of some variants have been reported [4], most genomic loci have not been carefully studied and little is known regarding target genes, pathways, or mechanisms of action. Multiple reports suggest that GWAS variants are overrepresented in sequences that regulate gene expression [3, 5, 6]. Several studies have shown enrichment for GWAS variants in cell- and tissue-specific regulatory elements [3, 5, 7, 8].

To aid interpretation of GWAS variants in the context of gene regulation, researchers have used large-scale mapping data for enhancers and other regulatory elements from ENCODE [9], Roadmap Epigenomics [6], and BLUEPRINT [10]. Several webtools, such as HaploReg [11], RegulomeDB [12], and others (reviewed in [13]), have been developed to help researchers link these data to individual variants. However, these methods do not include high-dimensional ENCODE data from contemporary technologies, such as Hi-C [14], or expanded expression quantitative trait locus (eQTL) data from large consortia, such as the Genotype-Tissue Expression Project (GTEx) [15] or the eQTLGen project [16]. Gathering relevant information from many different data sources and linking the data to individual genetic variants can be challenging in terms of computational resources, data processing, quality control, and reproducibility.

## Results

To address this issue and provide researchers with a state-of-the-art web tool for variant annotation that includes these updated resources, we developed FORGEdb (https://forgedb.cancer.gov/, Table 1). FORGEdb incorporates a range of datasets covering three broad areas relating to gene regulation: regulatory elements, transcription factor (TF) binding, and target genes. First, using genome-wide epigenomic track data from ENCODE [9], Roadmap Epigenomics [6], and BLUEPRINT [10] consortia, FORGEdb links SNPs with data for candidate regulatory elements (e.g., enhancers, promoters and other regulatory element classes). Specifically, FORGEdb annotates variants for overlap with DNase I hotspots, histone mark broadPeaks, and chromatin states across a wide range of cell and tissue types. Second, within these candidate regulatory elements, FORGEdb integrates SNPs with transcription factor (TF) binding data via (a) overlap with TF motifs and (b) SNP-specific Contextual Analysis of TF Occupancy (CATO) scores, which provide a complementary line of evidence for TF binding computed from allele-specific TF occupancy data measured by DNase I footprinting [17]. Third, FORGEdb links SNPs to target genes by providing (a) the overlap between SNPs and enhancer-to-promoter looping regions (or other looping regions) using Activity-By-Contact (ABC) data [18] and (b) allele-specific expression quantitative trait locus (eQTL) annotations using large-scale data from GTEx [15] and eQTLGen [16]. In addition, FORGEdb includes annotations from datasets that aid interpretation of protein-coding changes. Specifically, it includes allele-specific Combined Annotation Dependent Depletion (CADD) scores, which measure the deleteriousness of SNPs using experimental data and simulated mutations [19]. Moreover, FORGEdb includes the latest sequence conservation data from the Zoonomia project [20] and ENCODE4 CRISPR (clustered regularly interspaced short palindromic repeats) regulatory element single guide RNA (sgRNA) sequences and other data [21]. By amalgamating these datasets into a single resource, FORGEdb offers an expanded set of annotations and a more comprehensive evaluation of individual variants beyond what is provided by other commonly used webtools (Table 1) [11–13].

**Table 1:** A comparison of features across FORGEdb, HaploReg and RegulomeDB

	**FORGEdb**	**HaploReg**	**RegulomeDB**
Roadmap chromatin states	**Yes**	**Yes**	**Yes**
TF motifs	**Yes**	**Yes**	**Yes**
SNP scoring system	**Yes**	No	**Yes**
Roadmap DNase-seq	**Yes**	**Yes**	No
Roadmap H3 histone mark data	**Yes**	**Yes**	No
SiPhy cons	No	**Yes**	No
caQTLs	No	No	**Yes**
3D genomic data (ABC Hi-C-based data)	**Yes**	No	No
CADD v1.6 data across different alleles	**Yes**	No	No
GTEx v8 allele-specific association data	**Yes**	No	No
eQTLGen allele-specific association data	**Yes**	No	No
BLUEPRINT DNase-seq	**Yes**	No	No
Allele-specific TF binding data (CATO)	**Yes**	No	No
Zoonomia allele-specific conservation data	**Yes**	No	No
ENCODE4 regulatory element CRISPR sgRNAs	**Yes**	No	No

To summarize the regulatory annotations and prioritize genetic variants for functional validation, we created a new scoring system for SNPs, combining all annotations relating to gene regulation into a single score called a FORGEdb score. Our objective was to create scores that were accessible and readily interpretable to researchers while emphasizing transparency. In order to ensure that no single annotation or dataset would dominate or skew the scoring system, leading to bias, we adopted a points-based method that evaluates each distinct experimental or technological approach separately. FORGEdb scores are computed based on the presence or absence of 5 independent lines of evidence for regulatory function:

1. DNase I hotspot, marking accessible chromatin (2 points)
2. Histone mark ChIP-seq broadPeak, denoting different regulatory states (2 points)
3. TF motif (1 point) and CATO score (1 point), marking potential TF binding
4. Activity-by-contact (ABC) interaction, indicating gene looping (2 points)
5. Expression quantitative trait locus (eQTL), demonstrating an association with gene expression (2 points)

These five lines of experimental evidence were chosen based on likelihood of providing an indication of biological function, availability of high-quality data across multiple tissues, and offering a distinct line of experimental information. To prioritize variants at a large scale for functional studies, it is critical to examine multiple different lines of experimental evidence to gain a comprehensive picture of potential biological mechanisms. It is also important to include datasets that have employed an agnostic approach and are not targeted to a specific gene(s) or genomic region(s) or limited to a single tissue type, which could introduce bias.

**Fig. 1:**
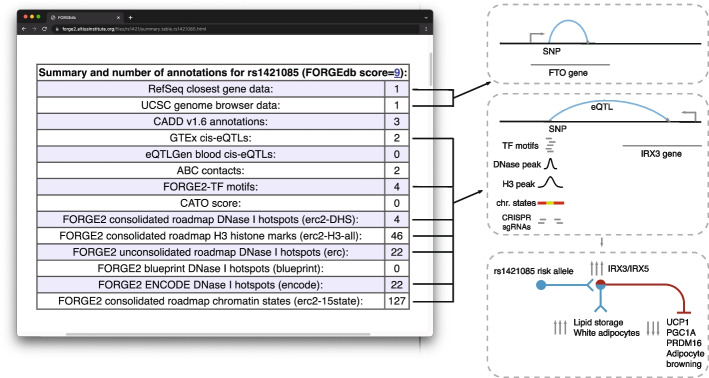
Example FORGEdb results for rs1421085. For this SNP, there is evidence for eQTL associations (with *IRX3* and *FTO*), chromatin looping (ABC interactions), overlap with significant TF motifs, and DNase I hotspot overlap, as well as overlap with histone mark broadPeaks. The only regulatory dataset that this SNP does not have evidence for is for CATO score (1 point). The resulting FORGEdb score for rs1421085 is therefore 9 = 2 (eQTL) + 2 (ABC) + 1 (TF motif) + 2 (DNase I hotspot) + 2 (histone mark ChIP-seq). Independent experimental analyses by Claussnitzer et al. have demonstrated a regulatory role for this SNP in the control of white vs. beige adipocyte proliferation via *IRX3/IRX5* [4]

FORGEdb scores were calculated by summing the number of points across all lines of evidence present for each SNP, and range between 0 and 10. A score of 9 or 10 suggests a large amount of evidence for functional impact, whereas 0 or 1 indicate a low amount of evidence. For example, there is evidence for eQTLs (for *IRX3* and *FTO*), chromatin looping, TF motifs, DNase I hotspots, and histone mark broadPeaks for rs1421085, a SNP previously identified for obesity [22] (Fig. 1). Together, these annotations provide strong evidence for a regulatory role for this SNP with a FORGEdb score of 9. This high FORGEdb score for rs1421085 is consistent with independent experimental analyses that have demonstrated a regulatory role for this SNP, with *IRX3* being a key target gene [4].

**Fig. 2:**
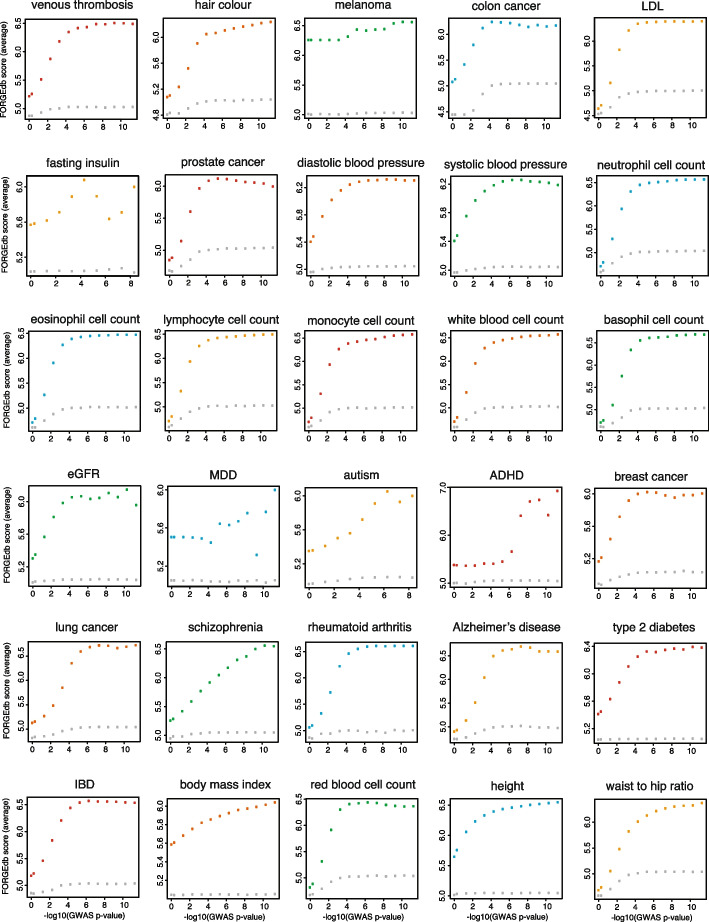
FORGEdb score (average, *y*-axis) versus GWAS -log10 (*p*-value) (*x*-axis) across 30 GWAS. Each colored point shows the FORGEdb score average across all GWAS SNPs at each *p*-value cutoff. Each grey point shows the FORGEdb score average across background SNPs (same minor allele frequency). From top left to bottom right: venous thrombosis (cor = 0.87), hair color (cor = 0.89), melanoma (cor = 0.95), colon cancer (cor = 0.77), LDL (cor = 0.81), fasting insulin (cor = 0.55), prostate cancer (cor = 0.78), diastolic blood pressure (cor = 0.82), systolic blood pressure (cor = 0.80), neutrophil cell count (cor = 0.82), eosinophil cell count (cor = 0.81), lymphocyte cell count (cor = 0.82), monocyte cell count (cor = 0.84), white blood cell count (cor = 0.83), basophil cell count (cor = 0.85), estimated glomerular filtration rate (eGFR, cor = 0.79), major depressive disorder (MDD, cor = 0.59), autism (cor = 0.96), attention deficit hyperactivity disorder (ADHD, cor = 0.89), breast cancer (cor = 0.79), lung cancer (cor = 0.90), schizophrenia (cor = 0.98), rheumatoid arthritis (cor = 0.86), Alzheimer’s disease (cor = 0.86), type 2 diabetes (cor = 0.88), inflammatory bowel disease (IBD, cor = 0.85), body mass index (cor = 0.96), red blood cell count (cor = 0.77), height (cor = 0.86), and waist-to-hip ratio (cor = 0.90)

To assess the potential utility of FORGEdb scores across different traits/diseases analyzed by GWAS, we obtained summary statistics from published studies of 30 traits/diseases (Methods) [2, 23–45] and evaluated the correlation between FORGEdb scores and the ranking of SNPs by association *p*-value in each GWAS. Specifically, we binned the SNPs according to their association -log10 *p*-value and estimated the mean FORGEdb score for each bin. Results revealed a significant positive correlation between mean FORGEdb score and ranked SNP bins across all 30 phenotypes, with more significant *p*-values corresponding to higher FORGEdb scores (Fig. 2 and Additional file 1, median correlation = 0.845, range 0.55 to 0.98). Further, to evaluate FORGEdb scores in fine-mapping studies, which can identify sets of variants more likely to be functional, we compared FORGEdb scores for variants from statistically-derived 95% credible sets with reported top SNPs from the same published study [46]. We discovered a significant overrepresentation of higher FORGEdb scores in the 95% credible sets (*t*-test *p*-value = 0.002). These findings demonstrate that FORGEdb scores correlate with GWAS associations and are significantly associated with GWAS 95% credible sets, and may therefore show utility for prioritizing SNPs across a wide range of human traits and diseases, from common traits such as brown hair color and height to complex diseases like schizophrenia and lung cancer.

**Fig. 3:**
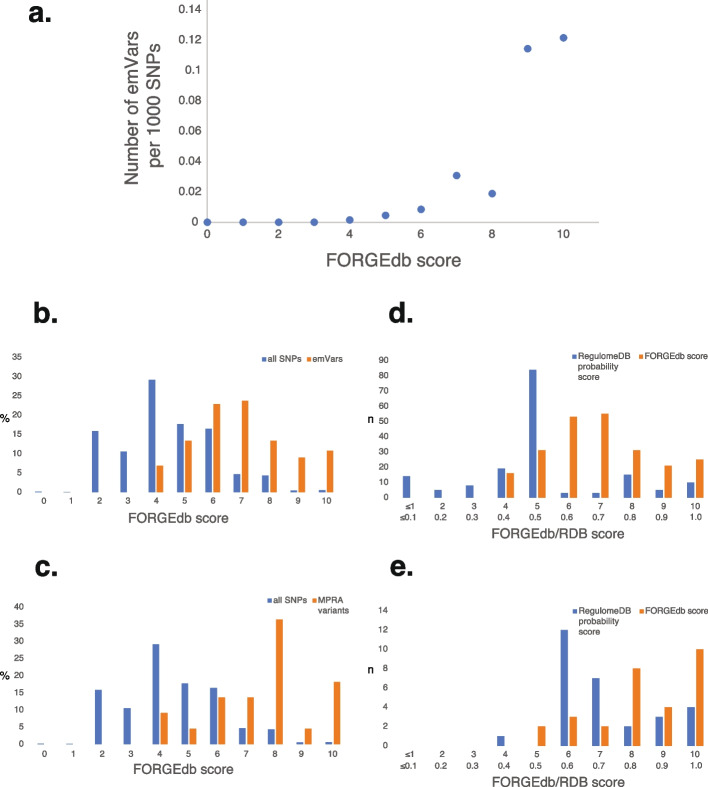
Variants identified from massively parallel reporter assays (MPRAs) are overrepresented in top FORGEdb scores. Shown here are (**A**) the number of expression-modulating variants (emVars) per 1000 SNPs (divisor, *y*-axis) for each FORGEdb score bin (0–10) (*x*-axis), (**B**) a histogram of FORGEdb scores for emVars (orange) and 37 million SNPs available in FORGEdb (blue), (**C**) a histogram of FORGEdb scores for *p* < 0.001 MPRA variants from Kircher et al. (orange) and 37 million SNPs available in FORGEdb (blue), (**D**) a bar chart of FORGEdb scores (orange) and RegulomeDB (RDB) scores for *p* < 0.000001 emVars (blue), and (**E**) a bar chart of FORGEdb scores (orange) and RegulomeDB scores for *p* < 0.05 MPRA variants for which both a FORGEdb score and a RegulomeDB score is available from Kircher et al. (blue)

To further assess the utility of FORGEdb scores in identifying potential functional variants, we examined the relationship between FORGEdb scores and expression-modulating variants (emVars), which are candidate functional variants prioritized from massively parallel reporter assays (MPRAs). We used emVar data from Tewhey et al. [47], who evaluated 39,487 variants using MPRAs, identifying 248 variants that had a high effect on gene expression. Comparing these 248 emVars with 37 million FORGEdb variants revealed a significant overrepresentation of emVars in higher FORGEdb scores (paired *t*-test *p*-value = 0.005, Fig. 3a, b). This suggests that variants with higher FORGEdb scores may more likely be functional and that FORGEdb scores are likely well-suited for prioritizing variants in MPRAs and other massively parallel experiments. Moreover, emVars exhibited significantly higher FORGEdb scores than 39,487 candidate MPRA variants from the same study (paired *t*-test *p*-value = 0.004), suggesting that FORGEdb scores add further information not present in previous variant prioritization methods. Additional comparisons with saturation mutagenesis MPRA data from Kircher et al. [48] (Fig. 3c, paired *t*-test *p*-value = 0.00974) and RegulomeDB scores across both MPRA datasets (Fig. 3d, e) further support these findings and indicate potential utility for FORGEdb scores in variant prioritization efforts for MPRAs and other massively parallel experiments.

## Discussion

FORGEdb exhibits several strengths and limitations. Although FORGEdb contains data on TF motifs and CATO scores for allele-specific DNase-seq-based TF binding, it does not have data on chromatin accessibility quantitative trait loci (caQTL), which are a similar dataset present in RegulomeDB. Additionally, even though FORGEdb includes recent conservation scores from the Zoonomia project, it does not include information on sequence constraint from SiPhy, which is present in HaploReg. Despite these limitations, FORGEdb remains a valuable resource for researchers seeking a comprehensive and integrated platform to annotate SNPs and interpret functional elements in the genome, particularly within the context of gene regulation and allele-specific effects.

FORGEdb has several strengths. Leveraging many different annotations, as well as its own SNP scoring system, FORGEdb facilitates a comprehensive analysis of variants and their regulatory context. It utilizes different types of DNase-seq and histone mark data to provide a deeper understanding of genomic regulatory landscapes. An additional distinctive feature of FORGEdb is its integration of 3D genomic data, specifically ABC Hi-C-based data, which permits the exploration of complex chromatin interactions, as well as genome editing resources (CRISPR regulatory element sgRNAs). Furthermore, FORGEdb incorporates CADD scores, providing further information about the potential deleterious effects of variant alleles. CADD scores, along with CATO scores, and allele-specific association data from GTEx and eQTLGen enable researchers to explore allele-specific effects in the context of genomic functionality. Neither ABC nor CADD scores nor CRISPR sgRNAs are available in RegulomeDB or HaploReg. In addition, FORGEdb scores correspond with functional significance based on MPRA data and may potentially be more informative for evaluating functional significance than probability scores provided in RegulomeDB.

## Conclusions

In summary, FORGEdb is a new web-based tool to aid the interpretation and prioritization of genetic variants for experimental analysis. FORGEdb includes a number of features from novel technologies not available in commonly used webtools, providing a more comprehensive analysis of potential regulatory function [11–13]. All of these features are accessible via a simple, easy-to-use search engine that can be found at https://forgedb.cancer.gov/ and https://forge2.altiusinstitute.org/files/forgedb.html. Annotations from FORGEdb can be accessed from https://ldlink.nih.gov/?tab=ldproxy, https://ldlink.nih.gov/?tab=ldassoc, https://ldlink.nih.gov/?tab=ldmatrix, and https://forge2.altiusinstitute.org/ [5, 49, 50].

## Methods

### Databases used in FORGEdb

FORGEdb standalone first annotates variants for positional overlap with DNase I hotspots, histone mark broadPeaks, and chromatin states across a wide range of cell and tissue types as implemented in FORGE2 [5]. Second, FORGEdb annotates variants for Activity-By-Contact (ABC) data (as implemented in Fulco et al.) [18], Contextual Analysis of TF Occupancy (CATO) scores as implemented by Maurano et al. [17], CADD scores as implemented by Rentzsch et al. [19], sequence conservation data from the Zoonomia project [20], TF motifs as implemented in FORGE2-TF (https://forge2-tf.altiusinstitute.org/ and https://analysistools.cancer.gov/forge2-tf/#/forge2-tf), significant eQTLs from GTEx [15] and eQTLGen [16], ENCODE4 regulatory element CRISPR sgRNAs from the ENCODE4 multi-center study computed via GuideScan2 [21, 51], and closest gene from RefSeq [52].

### FORGEdb scores

For all variants, we generated a FORGEdb score to reflect the extent of experimental evidence supporting possible functional significance. The objective was to ensure that FORGEdb scores were accessible to a wide array of researchers while emphasizing transparency and interpretability. A points-based system was applied to encompass a broad spectrum of experimental evidence from diverse data sources and to limit bias toward any particular line of evidence.

In creating the FORGEdb scores, we focused on datasets covering major areas of regulatory genomics with high-quality data across multiple tissues, identifying five key types of experimental evidence:

1. Chromatin accessibility. Evidence of chromatin accessibility, which is important for gene regulation, was assessed based on positional overlap with DNase I hotspots from the Roadmap Epigenomics consortium, ENCODE and BLUEPRINT, as analyzed in FORGE2 [5, 6, 9, 10]
2. Histone marks. Evidence for positional overlap with histone marks was assessed using broadPeak ChIP-seq data from the consolidated Roadmap H3-all dataset, which covers the 5 main histone marks analyzed across the main Roadmap tissue set (H3K4me1, H3K4me3, H3K36me3, H3K9me3, H3K27me3) [6]
3. Activity-by-contact (ABC) 3D genomics interactions. Evidence of ABC 3D genomics interactions, predictive of target gene looping, was assessed using positional overlap with ABC regions [18]
4. Differential gene expression. Evidence of allelic associations with gene expression were assessed using expression quantitative trait locus (eQTL) data from GTEx and eQTLGen [15, 16]
5. Transcription factors. Evidence of potential alteration of transcription factor binding was assessed by positional overlap with transcription factor (TF) motifs from FORGE2-TF (https://analysistools.cancer.gov/forge2-tf/#/forge2-tf and https://forge2-tf.altiusinstitute.org/) and Contextual Analysis of TF Occupancy (CATO) scores [17], which provide a measure of allele-specific associations with TF binding for a wide array of TFs

Equal weights (2 points each) were assigned to each line of evidence to prevent bias originating from any one approach. Resulting points were then added to provide a final FORGEdb score ranging from 0 to 10. When we applied this scoring system to 37 million variants, we observed an approximately normal distribution (Fig. 3b). We further validated the scoring system by assessing it against MPRA data, providing additional support for its alignment with functional significance.

### Allele-specific and regional data

FORGEdb provides a range of functional genomic annotations that can be categorized as based on positional overlap (e.g., the variant is located in a genomic region demarcated by the annotation) or variant-level features (e.g., allelic differences at the locus are associated with a particular feature). Among the regional overlap features, FORGEdb includes ABC data, CRISPR regulatory element sgRNAs, TF motifs, DNase I hotspots, and histone mark broadpeaks, offering insight into genomic context. Variant-level features, such as GTEx and QTLGen eQTL datasets, CATO scores, Zoonomia PhyloP scores, and CADD scores, provide allele-specific information. Collectively, these annotations in FORGEdb contribute to a comprehensive understanding of allele-specific effects and regional genomic context for individual SNPs.

### Accessing FORGEdb

FORGEdb is available via web browser (https://forgedb.cancer.gov/ and https://forge2.altiusinstitute.org/files/forgedb.html). A programmatic interface to FORGEdb has been developed via CRAN package LDlinkR (https://cran.r-project.org/web/packages/LDlinkR/index.html), and API instructions are at https://forgedb.cancer.gov/api-access. FORGEdb code is available under the MIT license from https://github.com/CBIIT/nci-webtools-dceg-forgedb and https://github.com/charlesbreeze/FORGEdb. FORGEdb constituent databases can be downloaded from https://github.com/CBIIT/nci-webtools-dceg-forgedb#building-and-hosting-the-api, and FORGEdb scores can be downloaded from Zenodo at https://doi.org/10.5281/zenodo.10067458.

### Example analysis

An example FORGEdb analysis is available at https://forgedb.cancer.gov/explore?rsid=rs12203592, with a complementary example at https://forge2.altiusinstitute.org/files/rs1421/summary.table.rs1421085.html. A description of FORGEdb scores is available at https://forgedb.cancer.gov/about/. A brief computational description of FORGEdb is available at https://github.com/CBIIT/nci-webtools-dceg-forgedb.

### Regenerating FORGEdb pages

To regenerate FORGEdb pages, we provide guidelines and code at https://github.com/CBIIT/nci-webtools-dceg-forgedb. Updated information on web server installation is available at https://github.com/CBIIT/nci-webtools-dceg-forgedb#building-and-hosting-the-api.

### Integration with summary statistics

Although FORGEdb does include blood cis-eQTL data from a large consortium, eQTLGen, offering additional information beyond GTEx, the FORGEdb webtool does not currently conduct colocalization analyses and thus does not compute the posterior probability of a variant affecting gene expression for a given GWAS. Regarding applications for summary statistics, we recommend modeling analysis in R using FORGEdb scores computed across over 37 million variants, which are scaled between 0 and 10 and are available for download at Zenodo at https://doi.org/10.5281/zenodo.10067458 (RSID.scores file), to facilitate integration and joint analysis of summary statistics and FORGEdb scores.

### Analysis of MPRA and GWAS data

To validate the utility of FORGEdb scores, we analyzed MPRA emVar data and publicly available GWAS data. For analysis of MPRA emVar data, we downloaded the SNP information from table S1 (39,478 ref/alt pairs tested by MPRA) and S2 (emVars) of Tewhey et al. [47]. We computed FORGEdb SNP scores for all 248 reported emVars and the other 39,478 SNPs evaluated in the manuscript. We then compared the FORGEdb scores for the emVars with the other evaluated SNPs and 37 million SNPs available in FORGEdb.

For analysis of Kircher et al. MPRA data, we downloaded the hg38 MPRA information from https://kircherlab.bihealth.org/satMutMPRA/ [48]. We then generated FORGEdb scores for variants with MPRA *p* < 0.001. We also integrated RegulomeDB scores with variants, which resulted in a reduced number of intersecting SNPs across all scores, so for this second comparison, we focused on variants at *p* < 0.05. We then plotted FORGEdb scores for the first set of variants alongside scores of background SNPs available in FORGEdb, and then plotted FORGEdb scores and RegulomeDB scores for the second set of variants.

For analysis of GWAS data across 30 disease/traits, we downloaded GWAS summary statistics from OpenGWAS [24] and other sources [2, 23–45]. Ethnicities analyzed in these GWAS include African American/Afro-Caribbean, East Asian, and European. For each GWAS, we computed FORGEdb scores across all variants and then computed the average score at different *p*-value thresholds. Published 95% credible sets for a coronary heart disease GWAS were obtained from van der Harst et al. [46]. Plotting and statistical analyses were conducted in R [53].

### Contact

For any questions or information contact c.breeze@ucl.ac.uk.

### Supplementary Information

**Additional file 1. **FORGEdb score (average, *y*-axis) versus GWAS -log10(*p*-value) (*x*-axis) across 30 GWAS, FORGE2 analysis. Each red point shows the FORGEdb score average across all GWAS SNPs at a each *p*-value cutoff. Each grey point shows the FORGEdb score average across background SNPs (FORGE2 linkage disequilibrium analysis, same minor allele frequency). FORGE2 SNP number requirements preclude background analysis for certain *p*-value thresholds in some of the GWAS. Order of panels: melanoma, monocyte cell count, diastolic blood pressure, systolic blood pressure, neutrophil cell count, eosinophil cell count, lymphocyte cell count, white blood cell count, basophil cell count, lung cancer, schizophrenia, estimated glomerular filtration rate, major depressive disorder, type 2 diabetes, body mass index, rheumatoid arthritis, height, waist-to-hip ratio, fasting insulin, red blood cell count, inflammatory bowel disease, Alzheimer’s disease, breast cancer, attention deficit hyperactivity disorder, autism, prostate cancer, LDL, hair color, colon cancer, and venous thrombosis.

**Additional file 2. **Review history.

**Additional file 3. **Instructions for hosting FORGEdb on a static file server.

## Data Availability

Data obtention ENCODE, BLUEPRINT, and Roadmap Epigenomics DNase I hotspot files, Roadmap Epigenomics BroadPeak Histone mark files, HMM Chromatin State files, and TF motif files were obtained as described previously [5, 54]. ABC, CADD, CATO, closest gene, ENCODE4 regulatory element CRISPR sgRNAs, eQTLs from GTEx and eQTLGen, and Zoonomia PhyloP scores were obtained from the respective studies [15–21, 52]. All these datasets are available for bulk download via the FORGEdb API and from Zenodo at https://doi.org/10.5281/zenodo.10067458. Instructions for downloading the FORGEdb constituent databases are at https://github.com/CBIIT/nci-webtools-dceg-forgedb#building-and-hosting-the-api. FORGEdb scores can be downloaded from Zenodo at https://doi.org/10.5281/zenodo.10067458. Source code and additional files FORGEdb is available via web browser (https://forgedb.cancer.gov/ and https://forge2.altiusinstitute.org/files/forgedb.html). A programmatic interface to FORGEdb has been developed via CRAN package LDlinkR (https://cran.r-project.org/web/packages/LDlinkR/index.html) and, additionally, API instructions are at https://forgedb.cancer.gov/api-access. FORGEdb source code is available under the MIT license from https://github.com/CBIIT/nci-webtools-dceg-forgedb, https://github.com/charlesbreeze/FORGEdb and https://doi.org/10.5281/zenodo.10067458 [55, 56]. Instructions for hosting FORGEdb on a static file server are available in Additional file 3.
