# Supplementary figures and images for: FORGEdb: a tool for identifying candidate functional variants and uncovering target genes and mechanisms for complex diseases
Source: Genome Biol. 2024 Jan 2;25:3. doi: 10.1186/s13059-023-03126-1 (PMC10763681; doi:10.1186/s13059-023-03126-1)

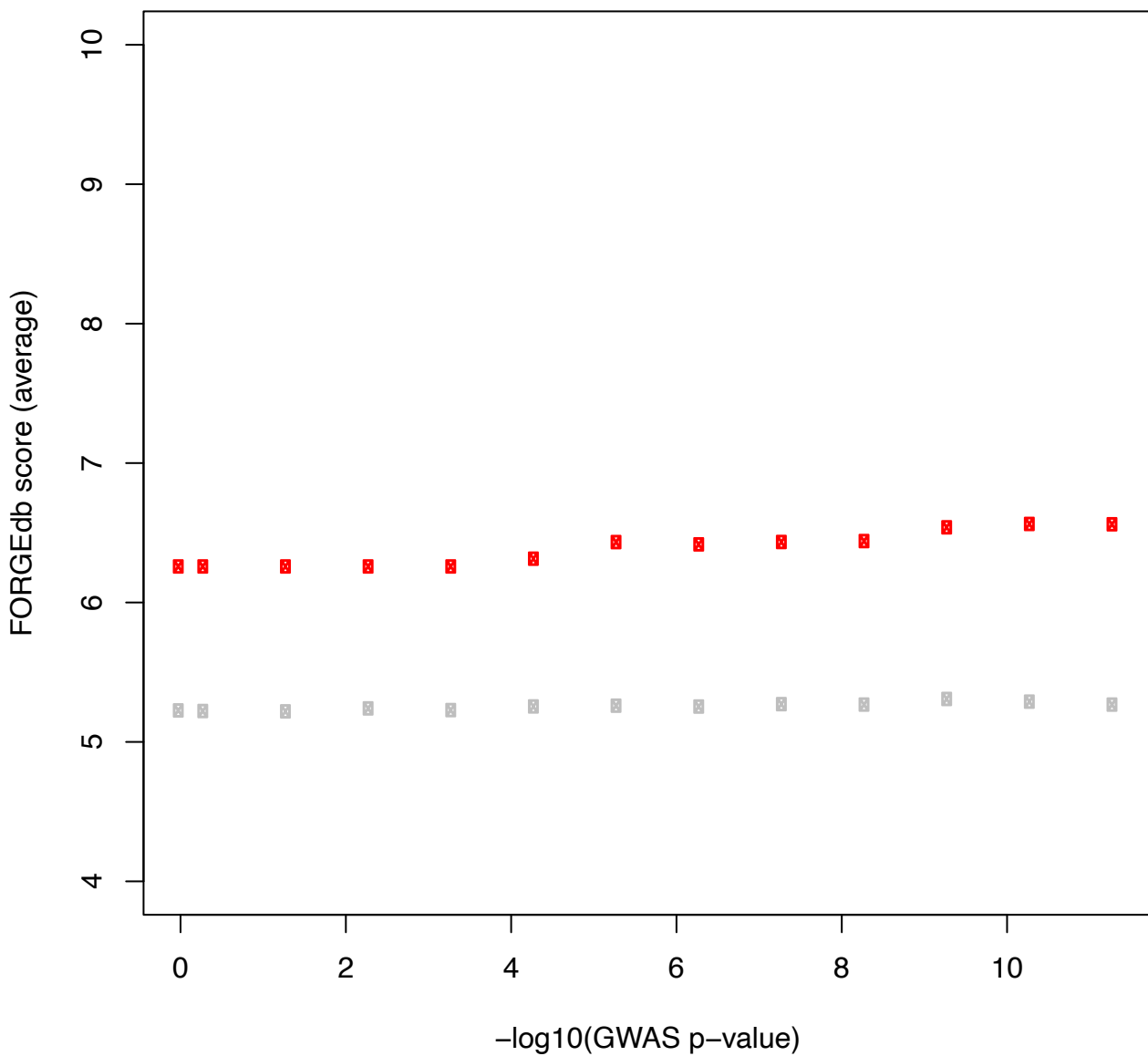

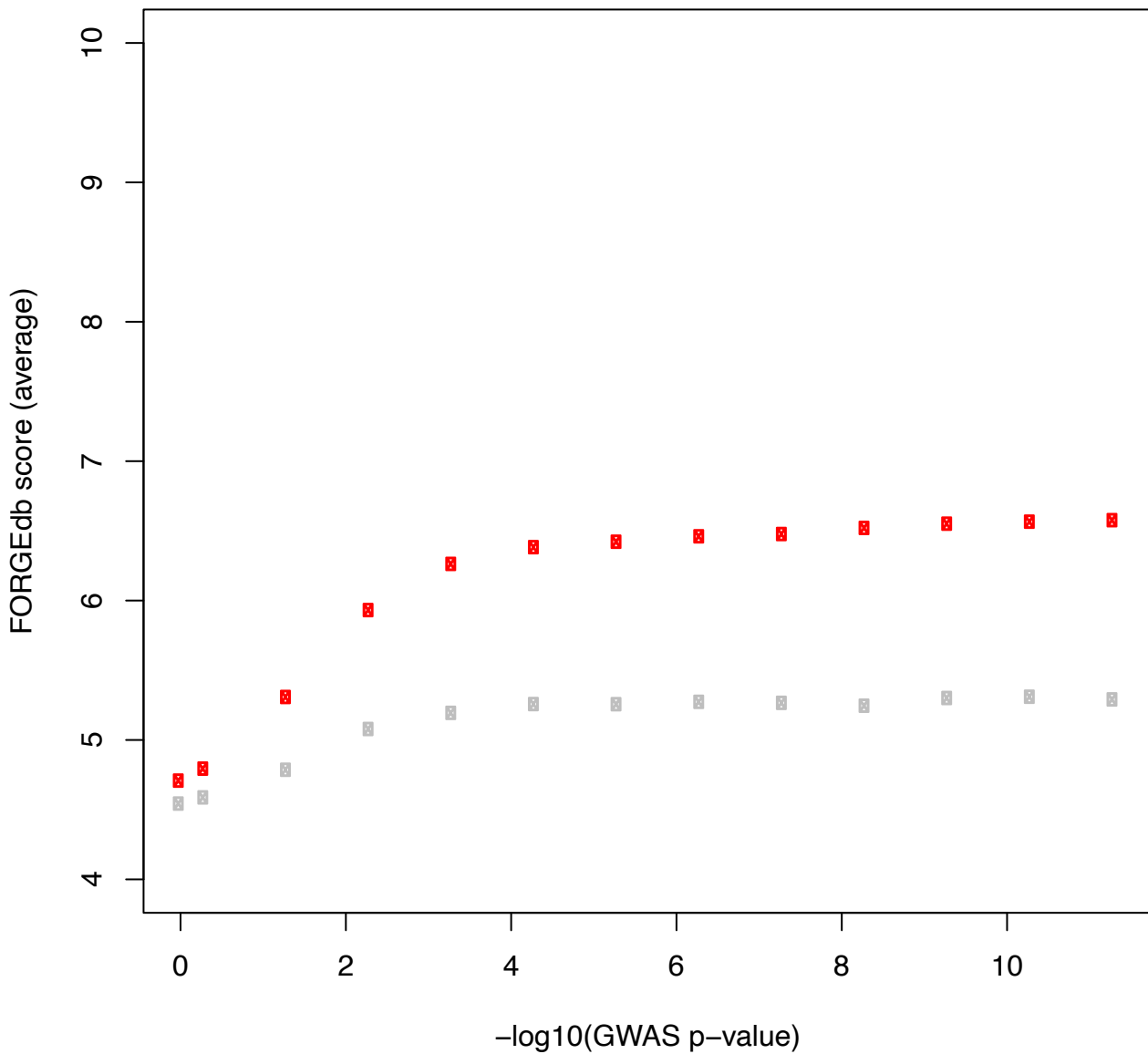

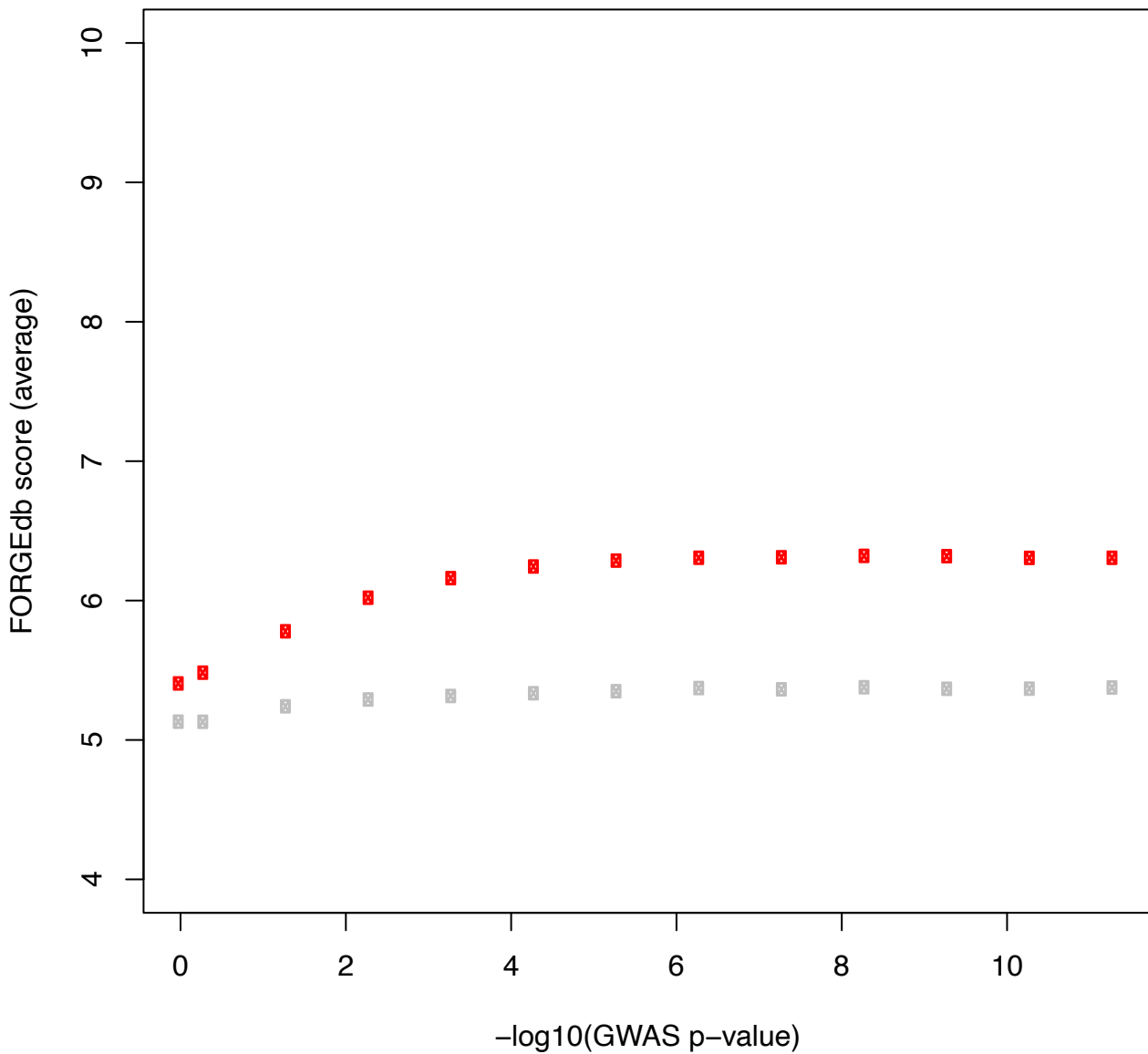

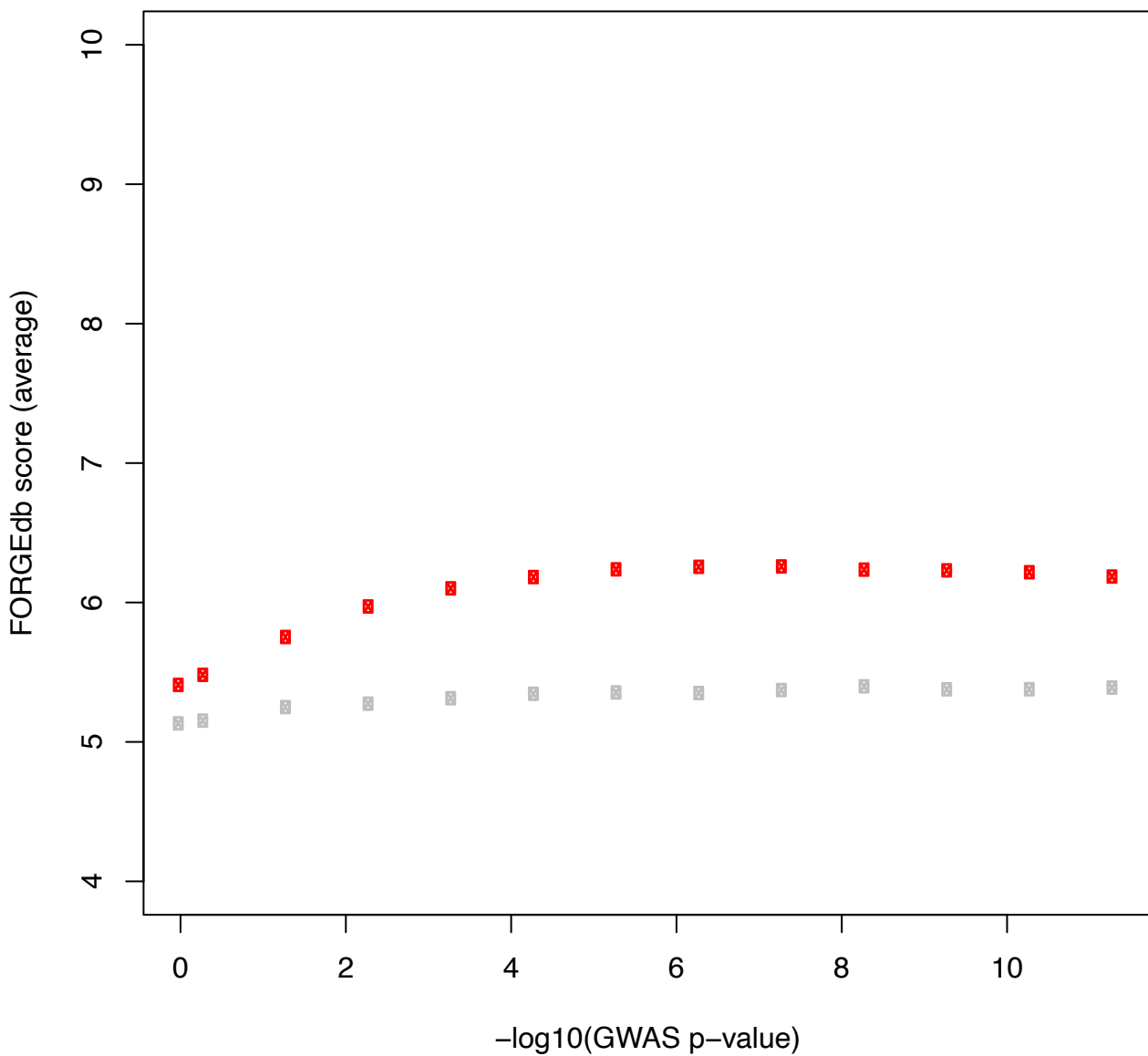

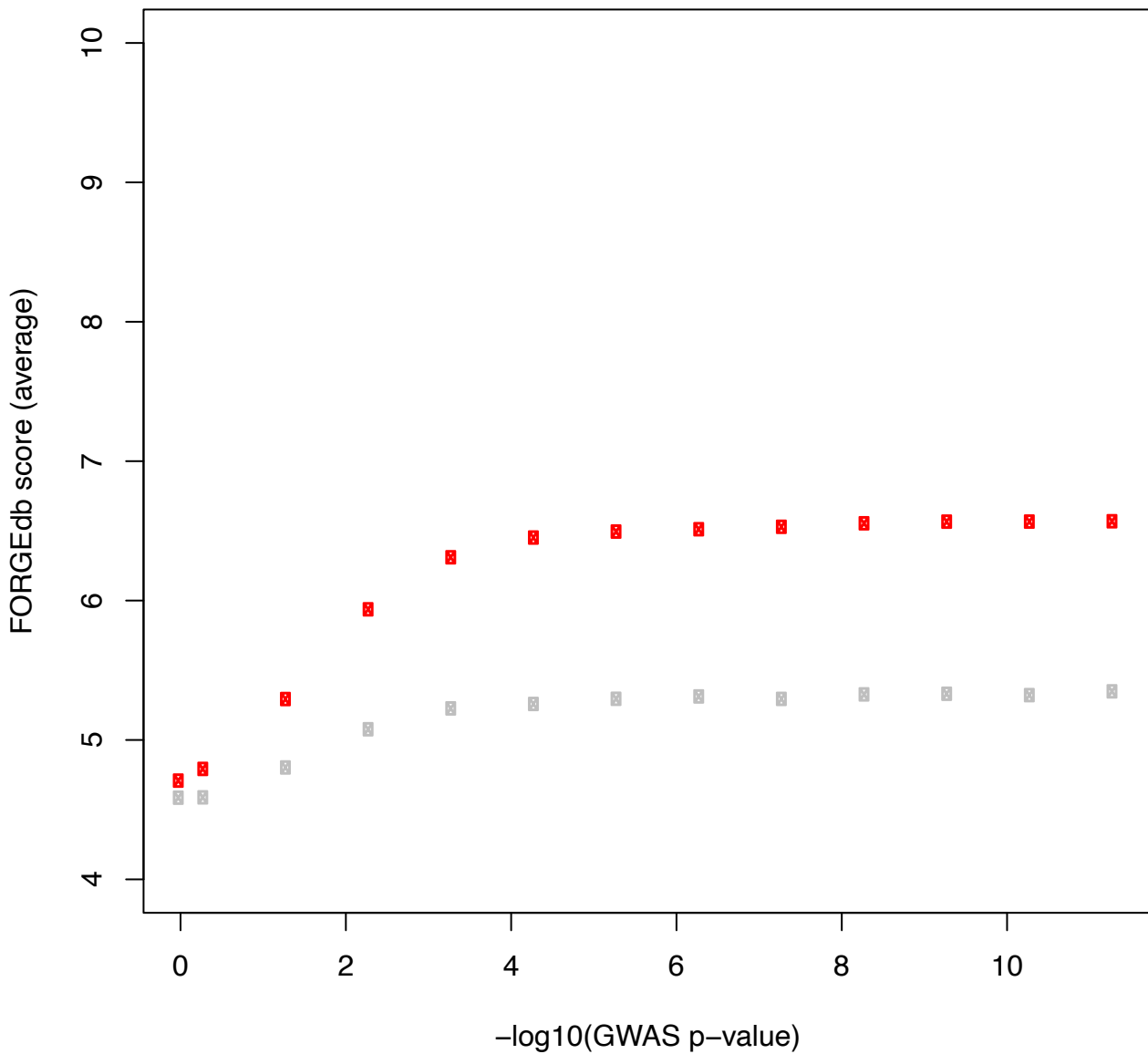

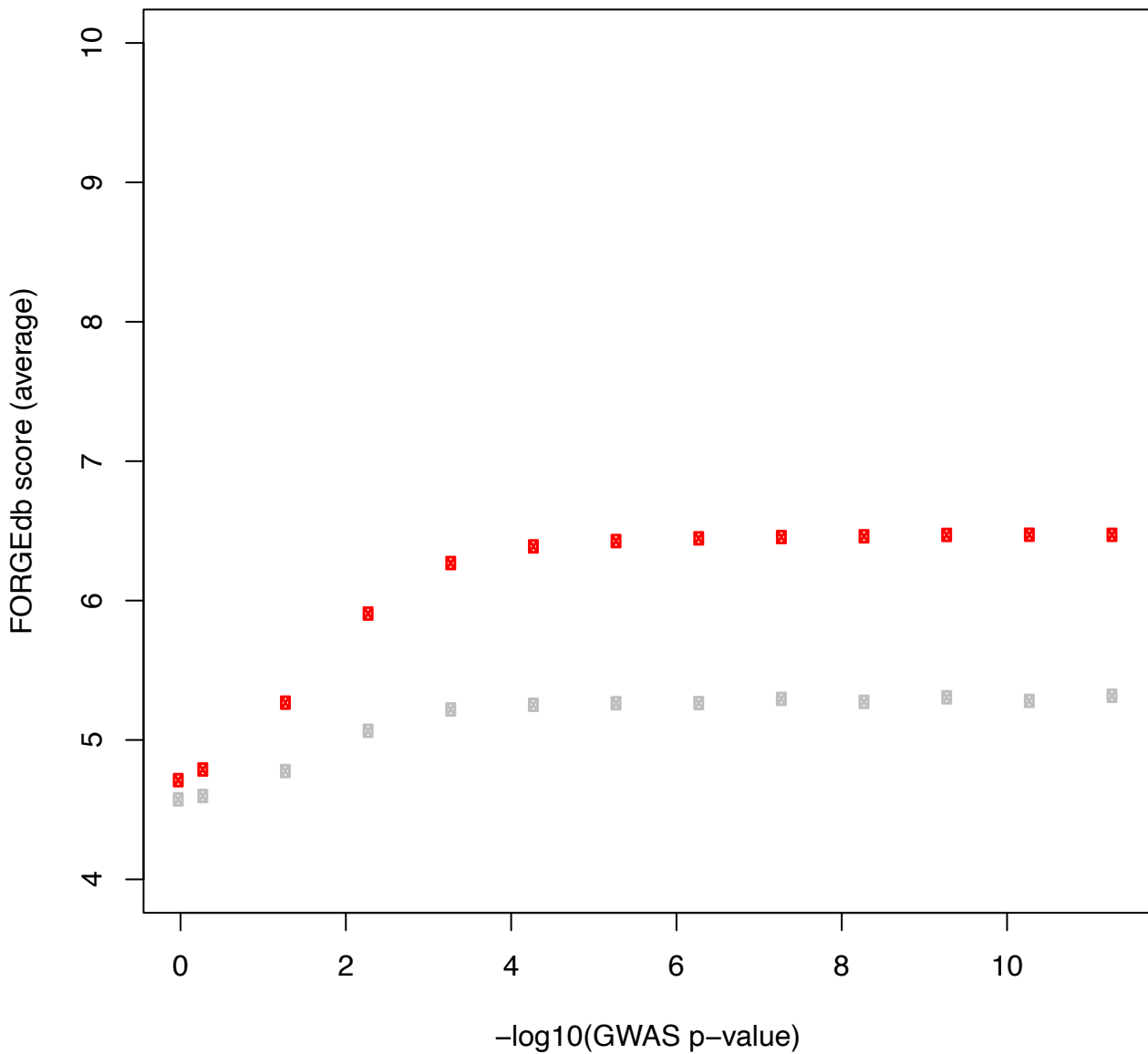

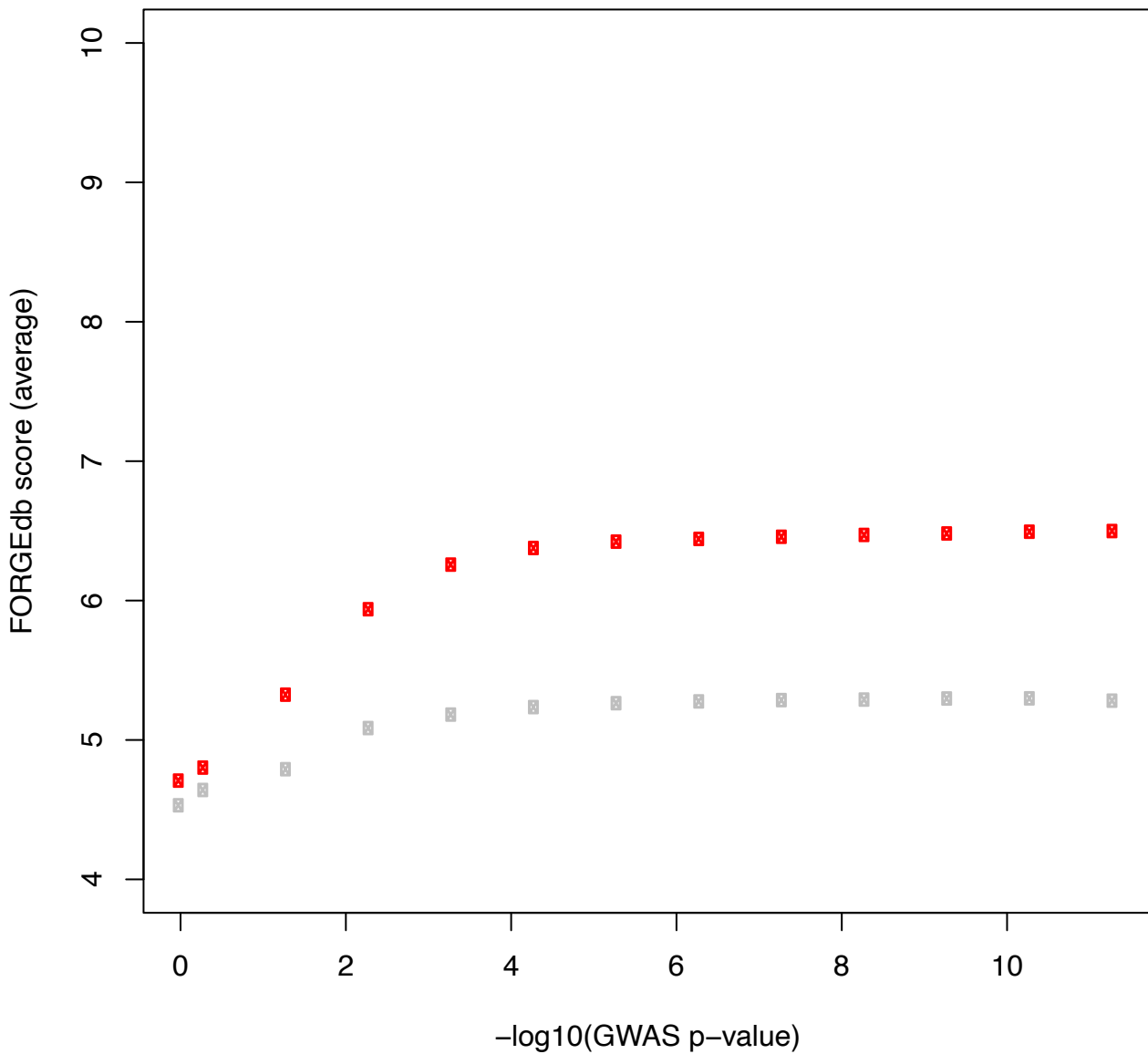

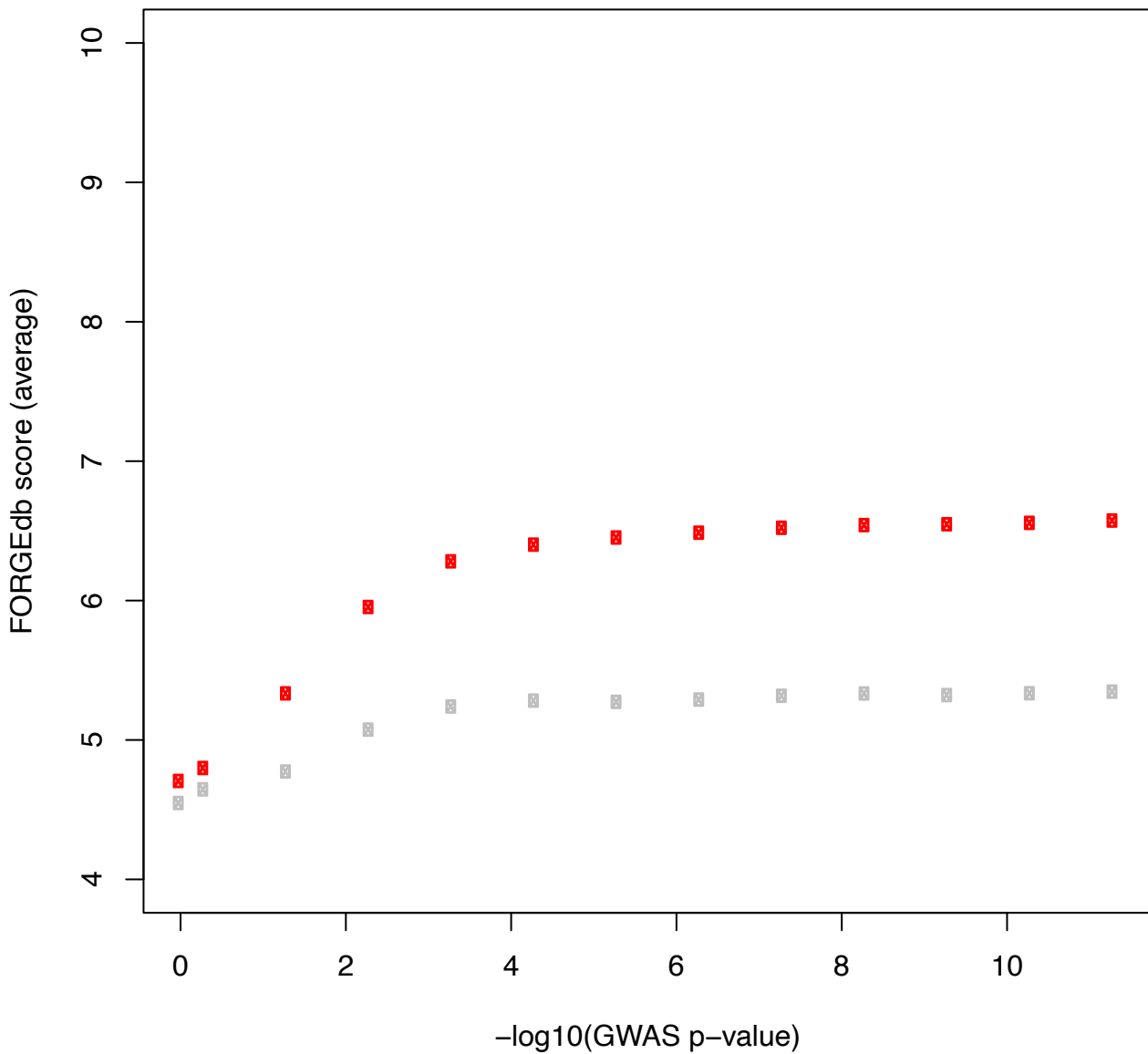

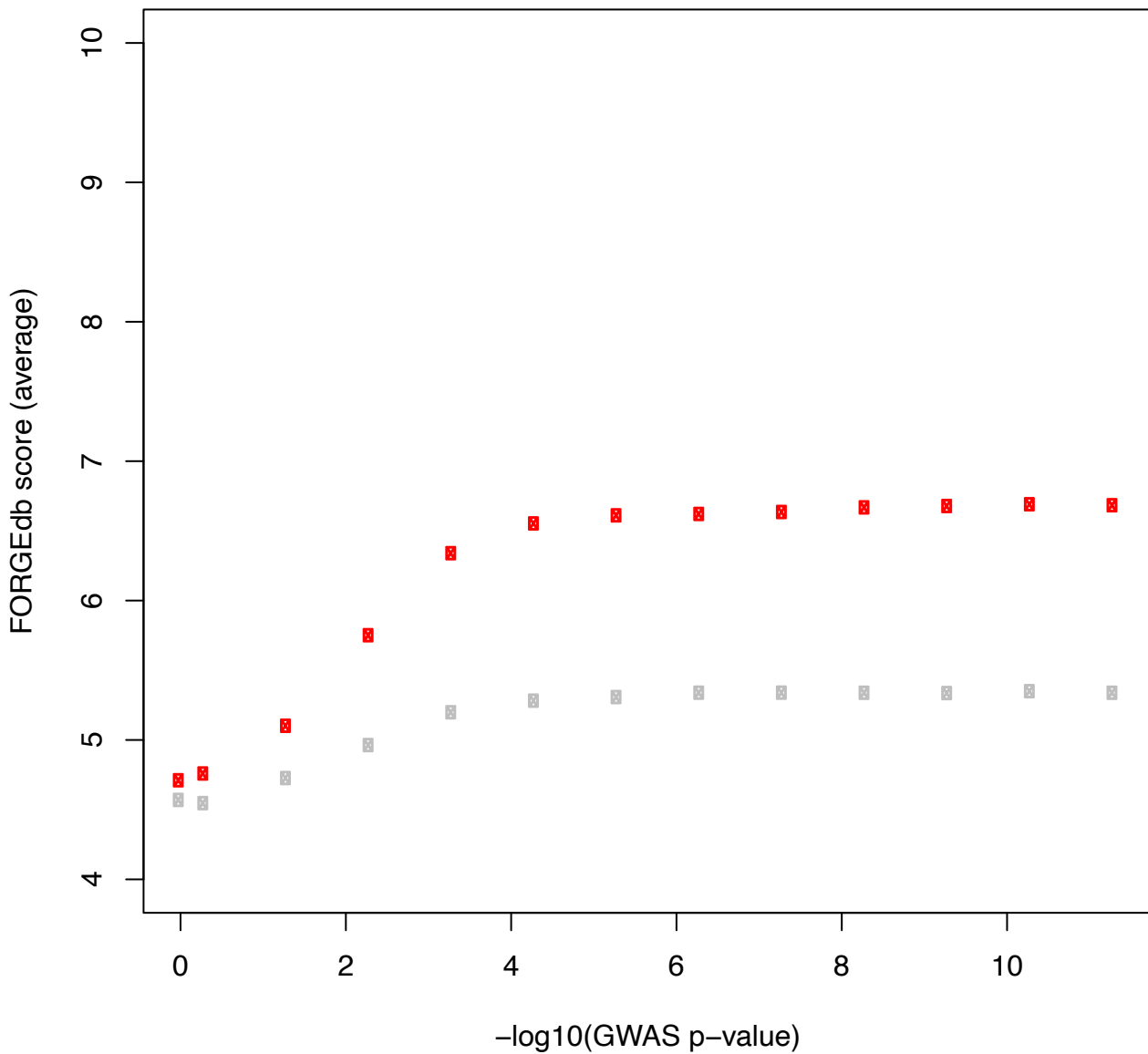

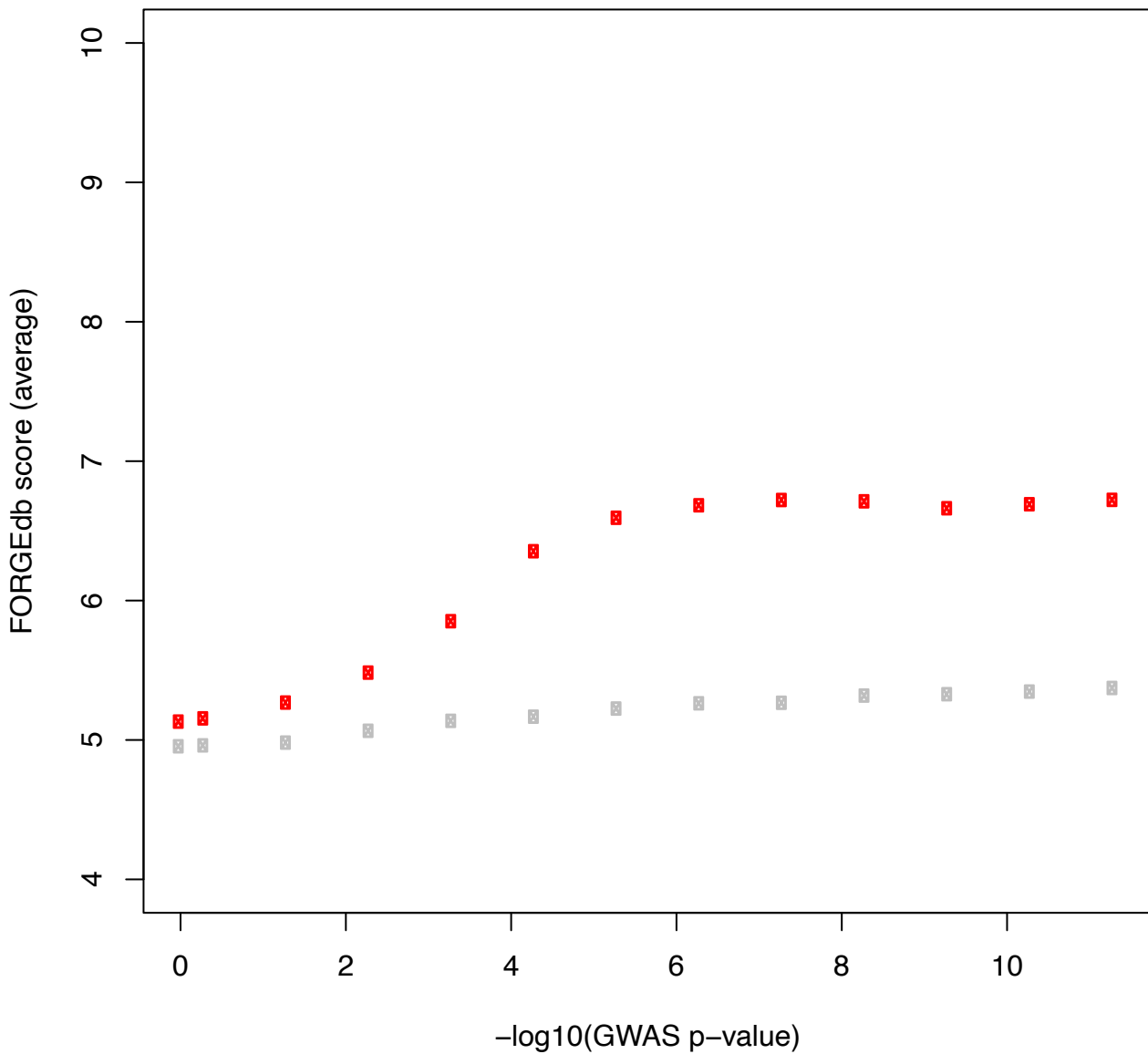

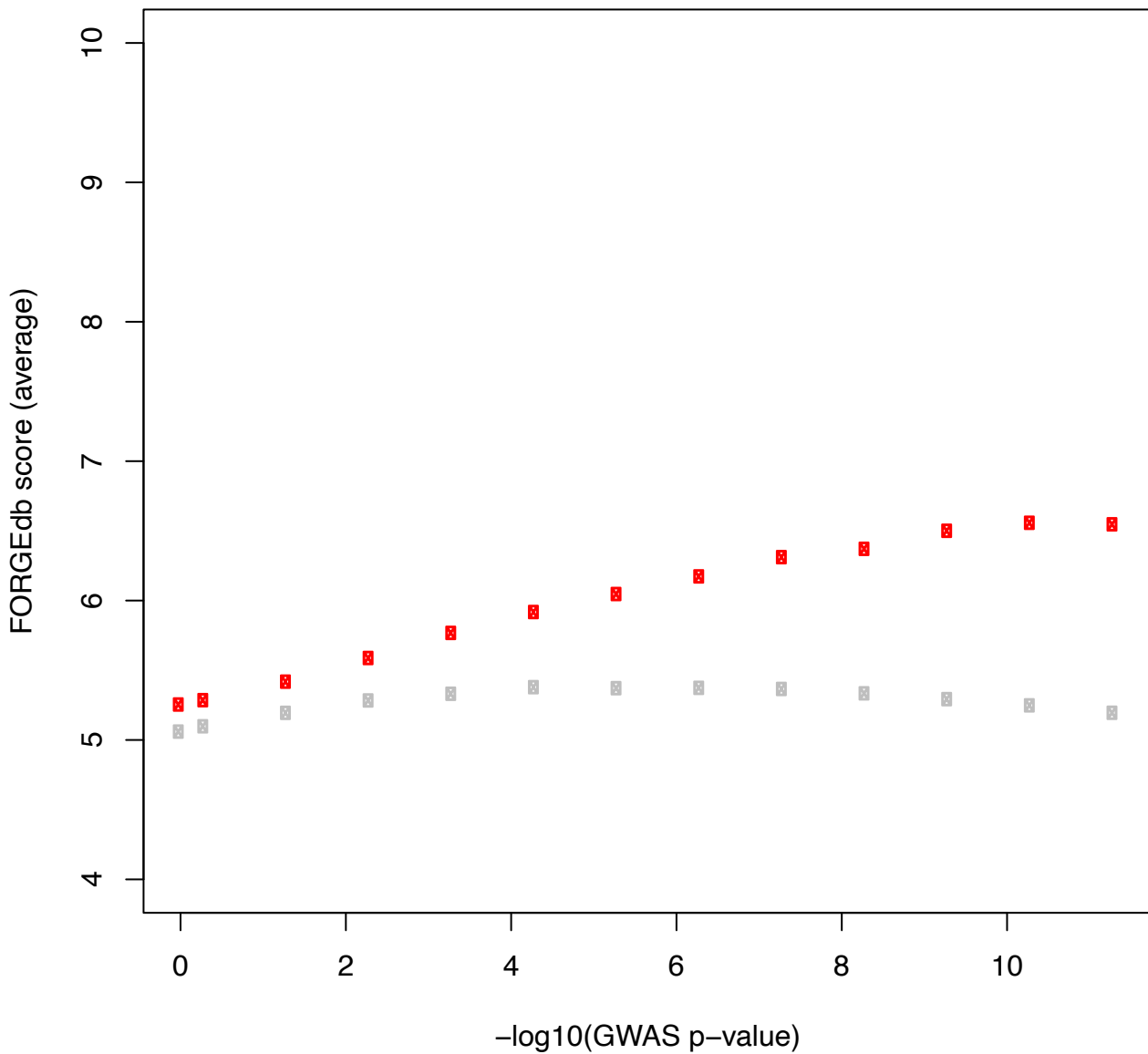

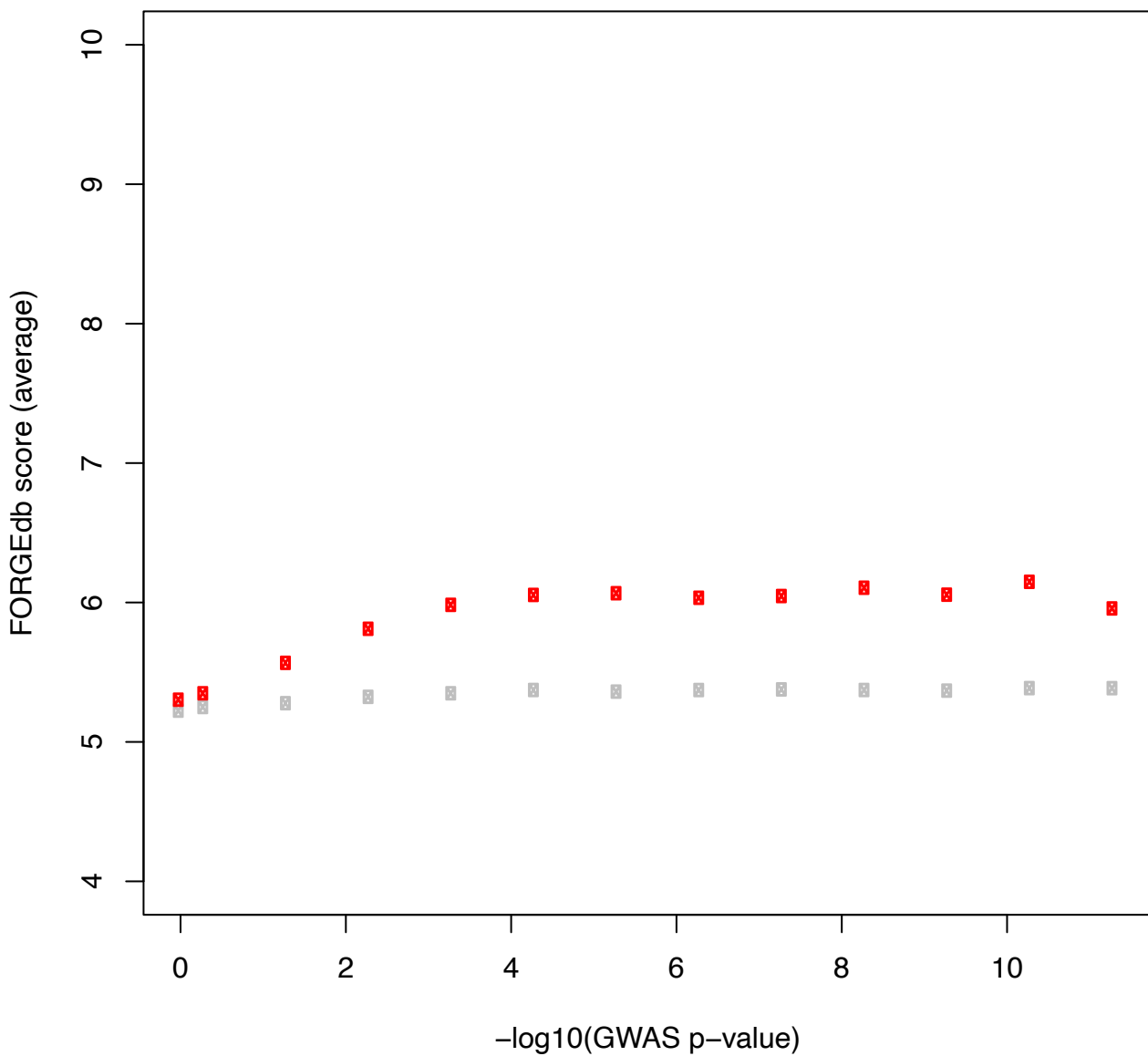

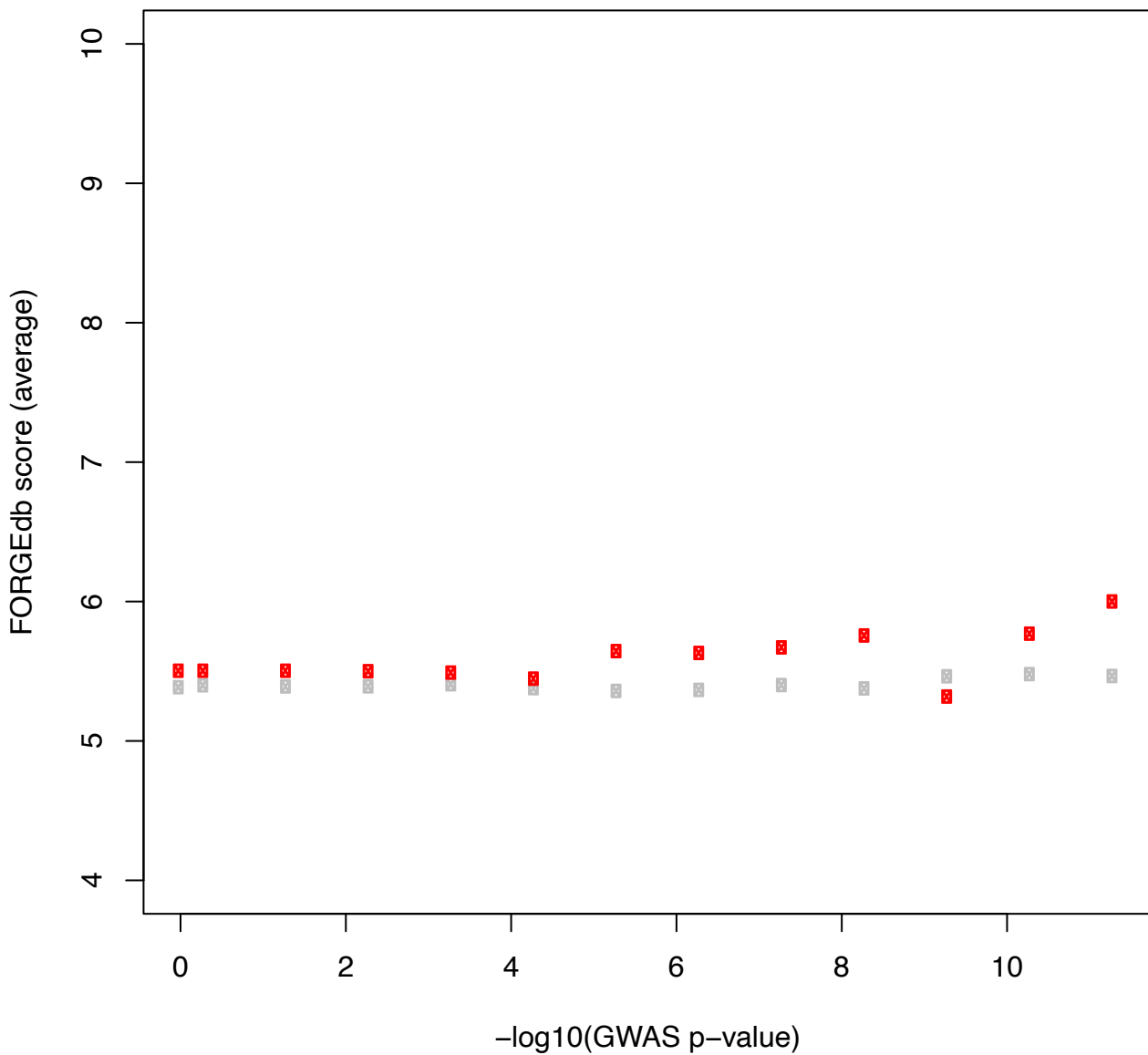

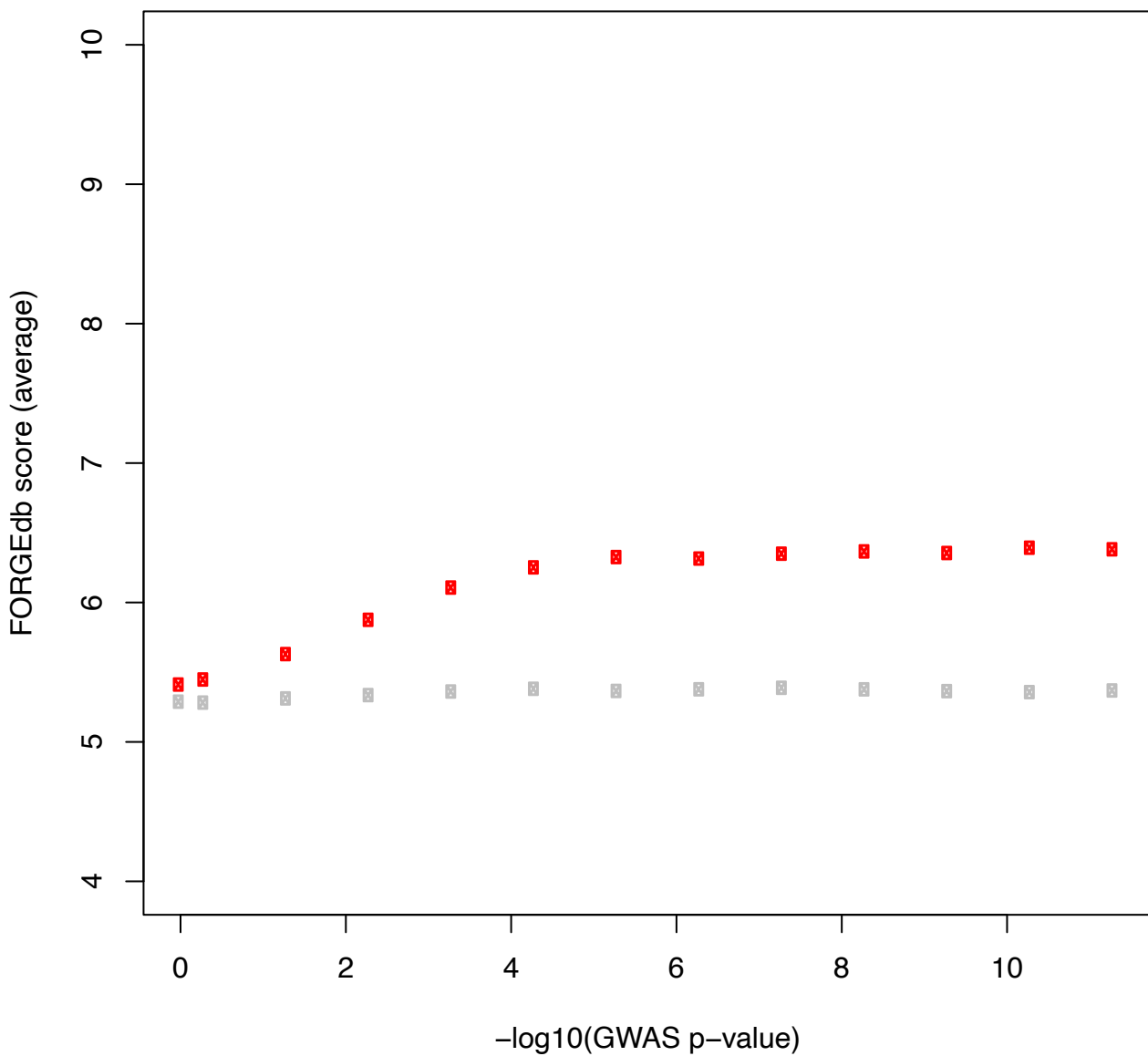

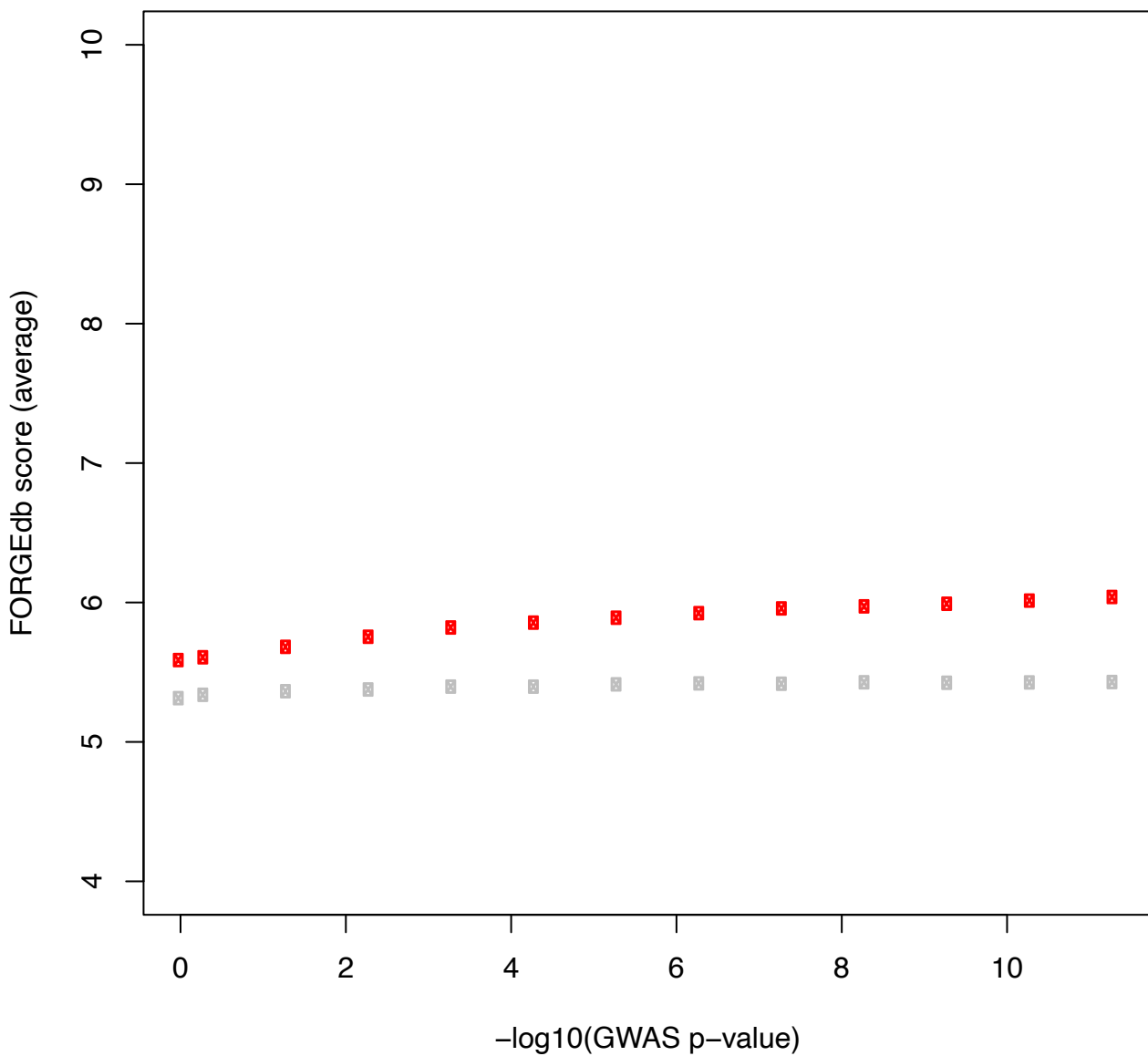

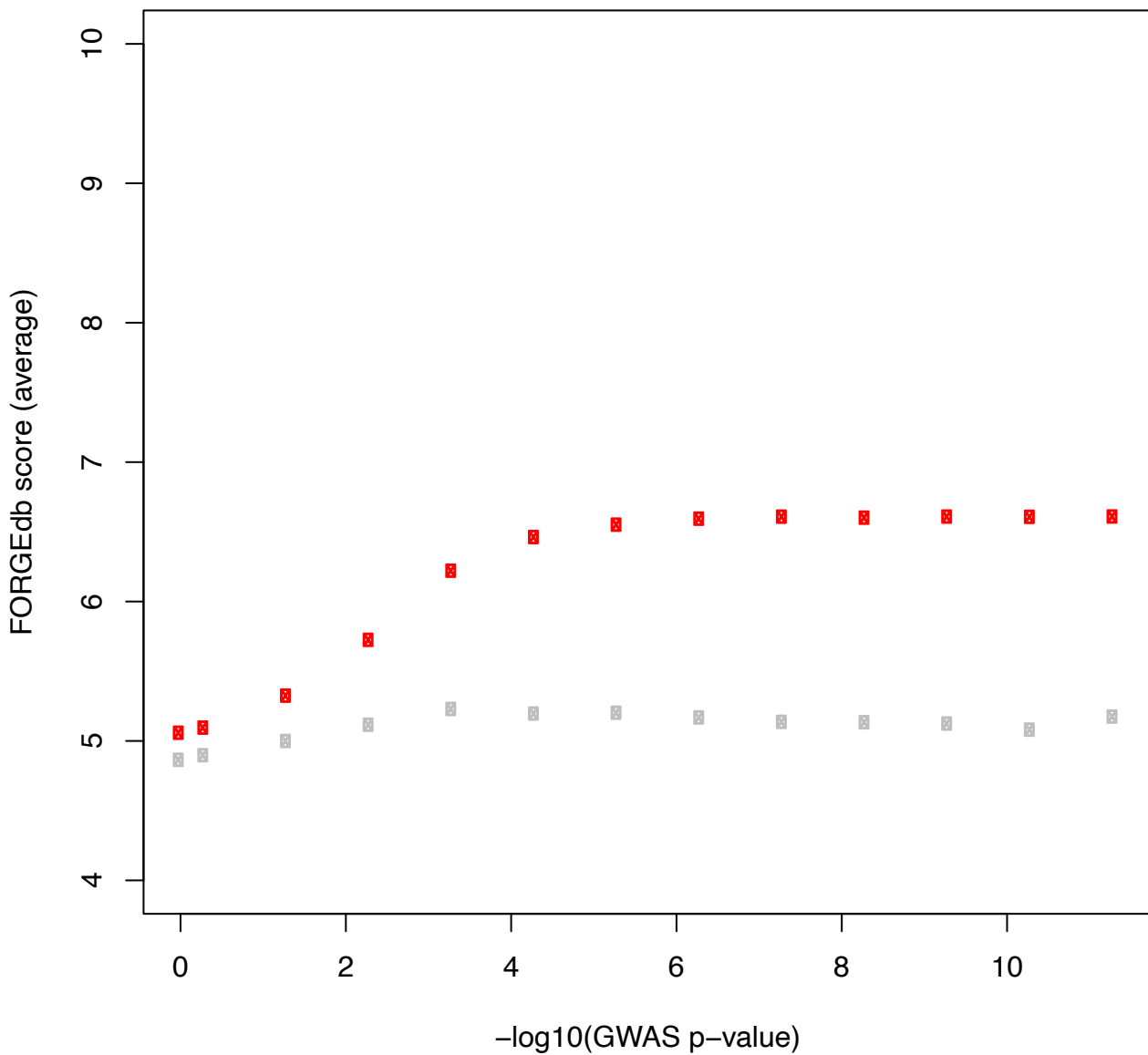

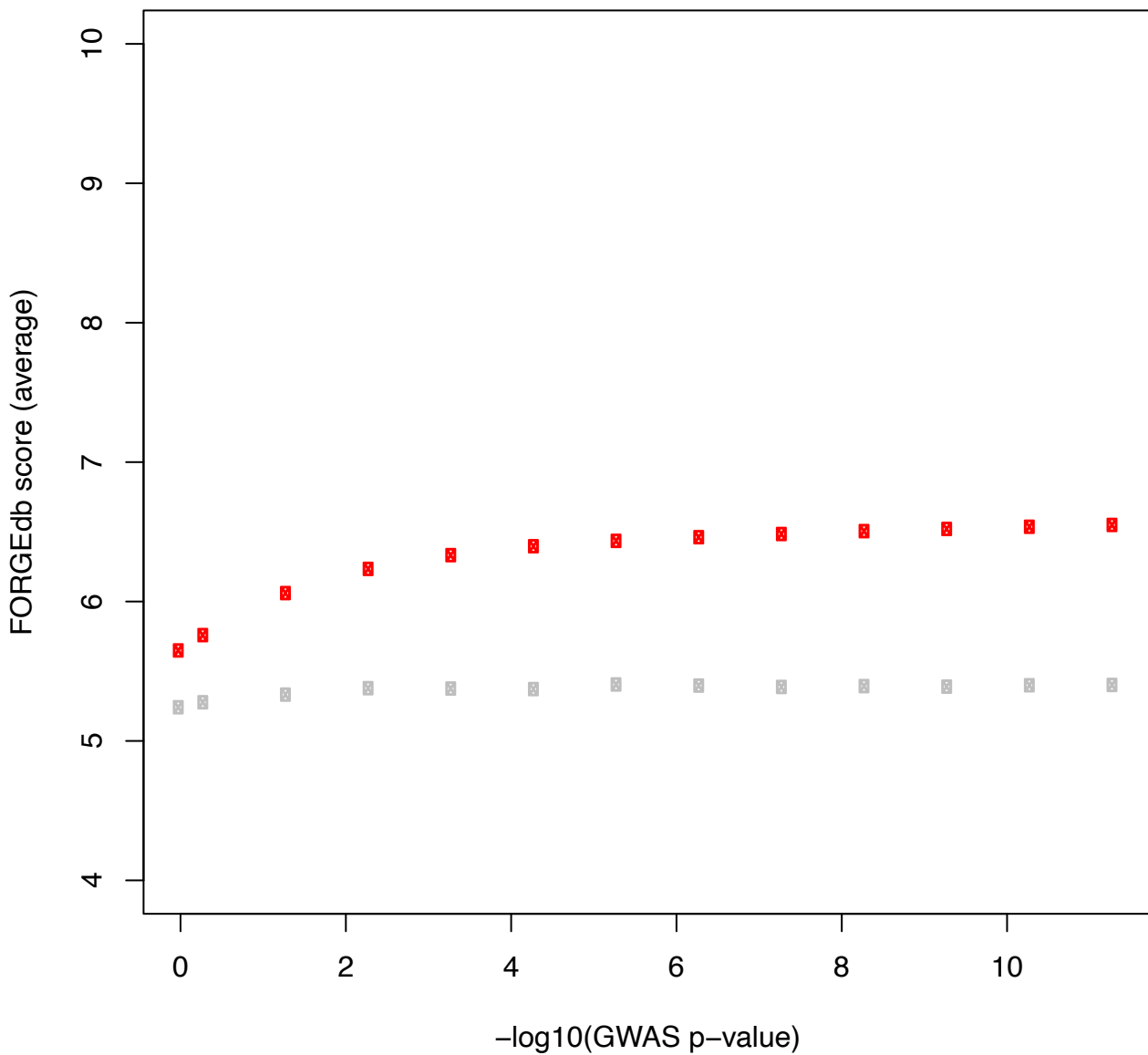

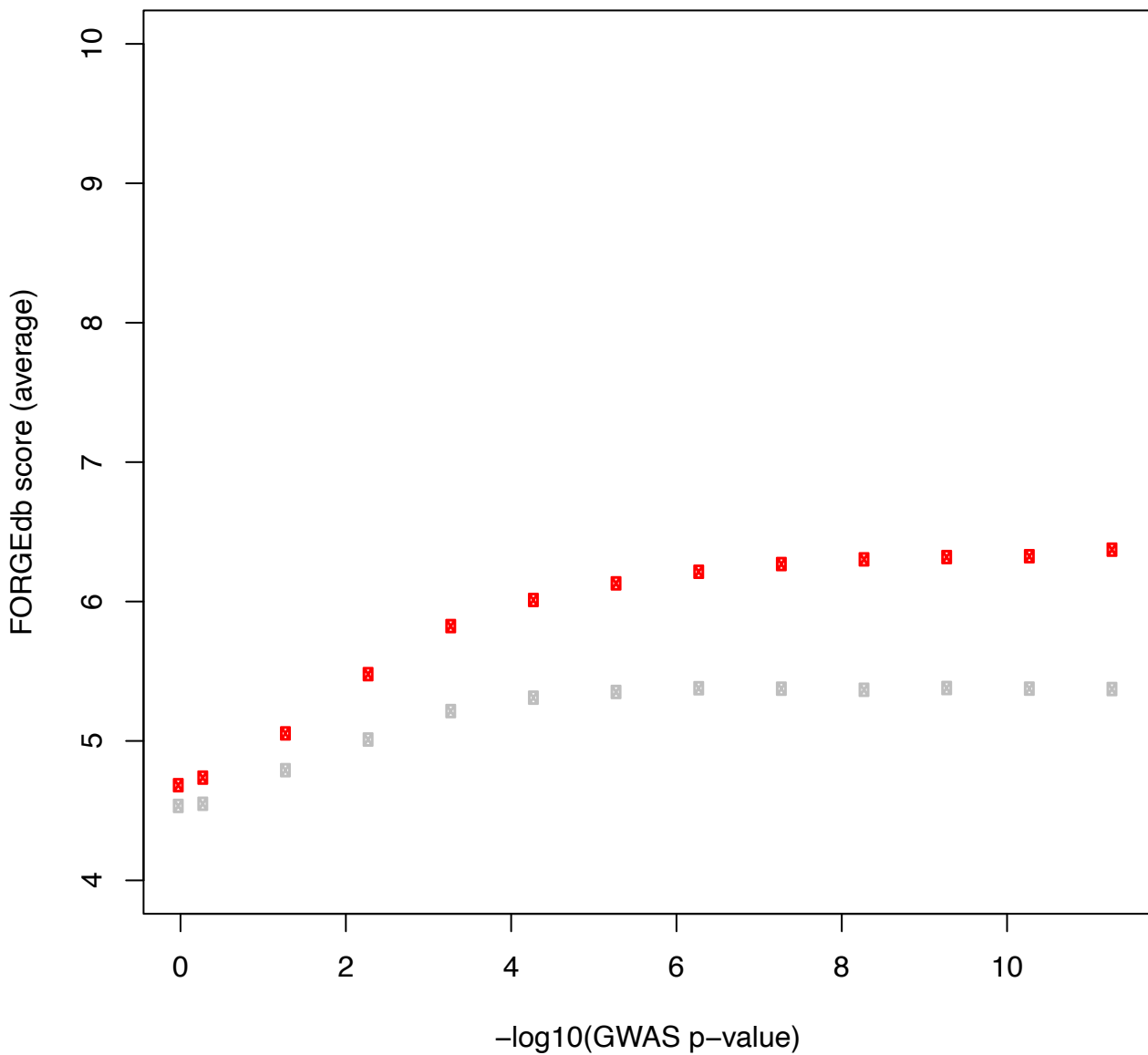

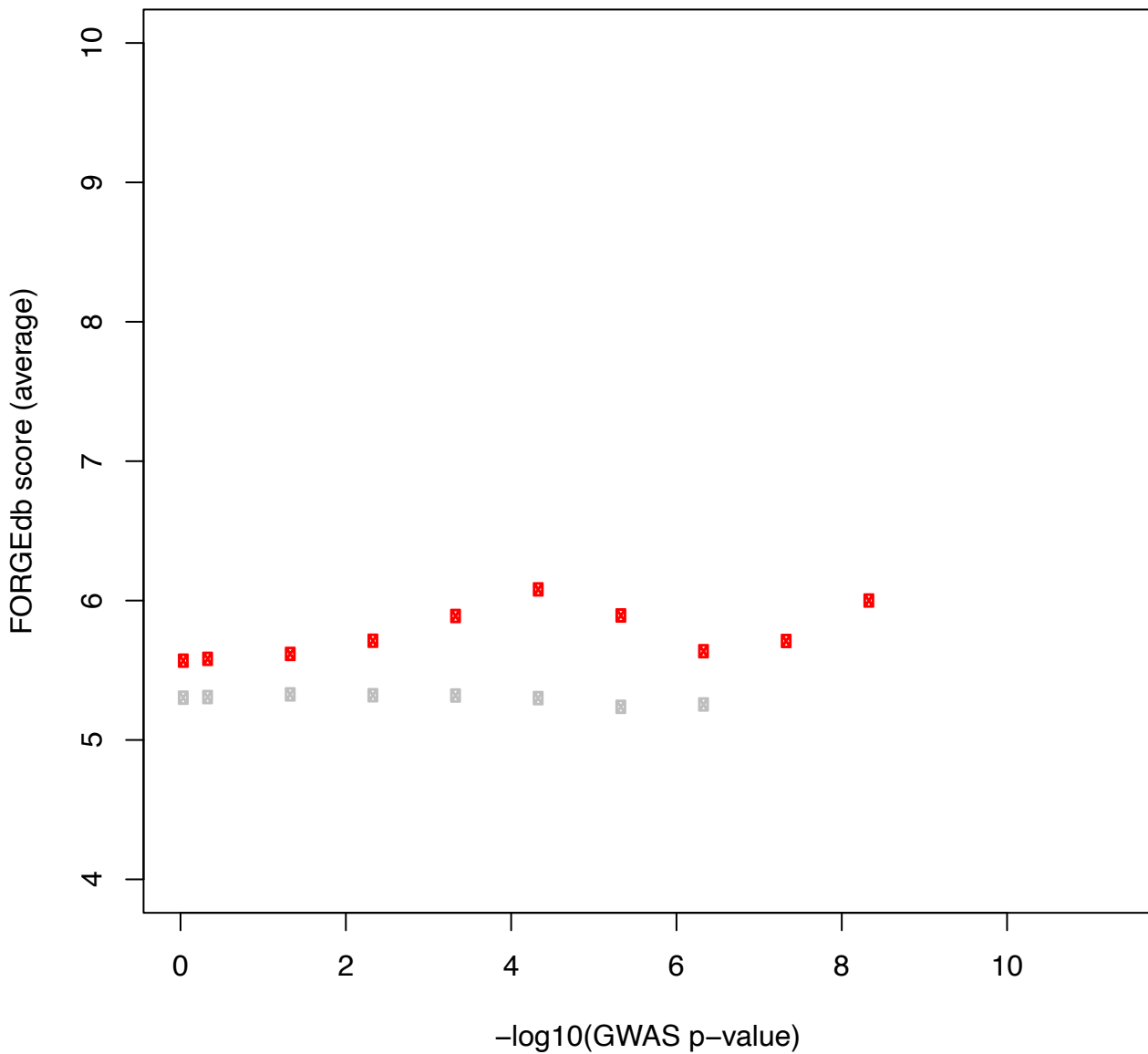

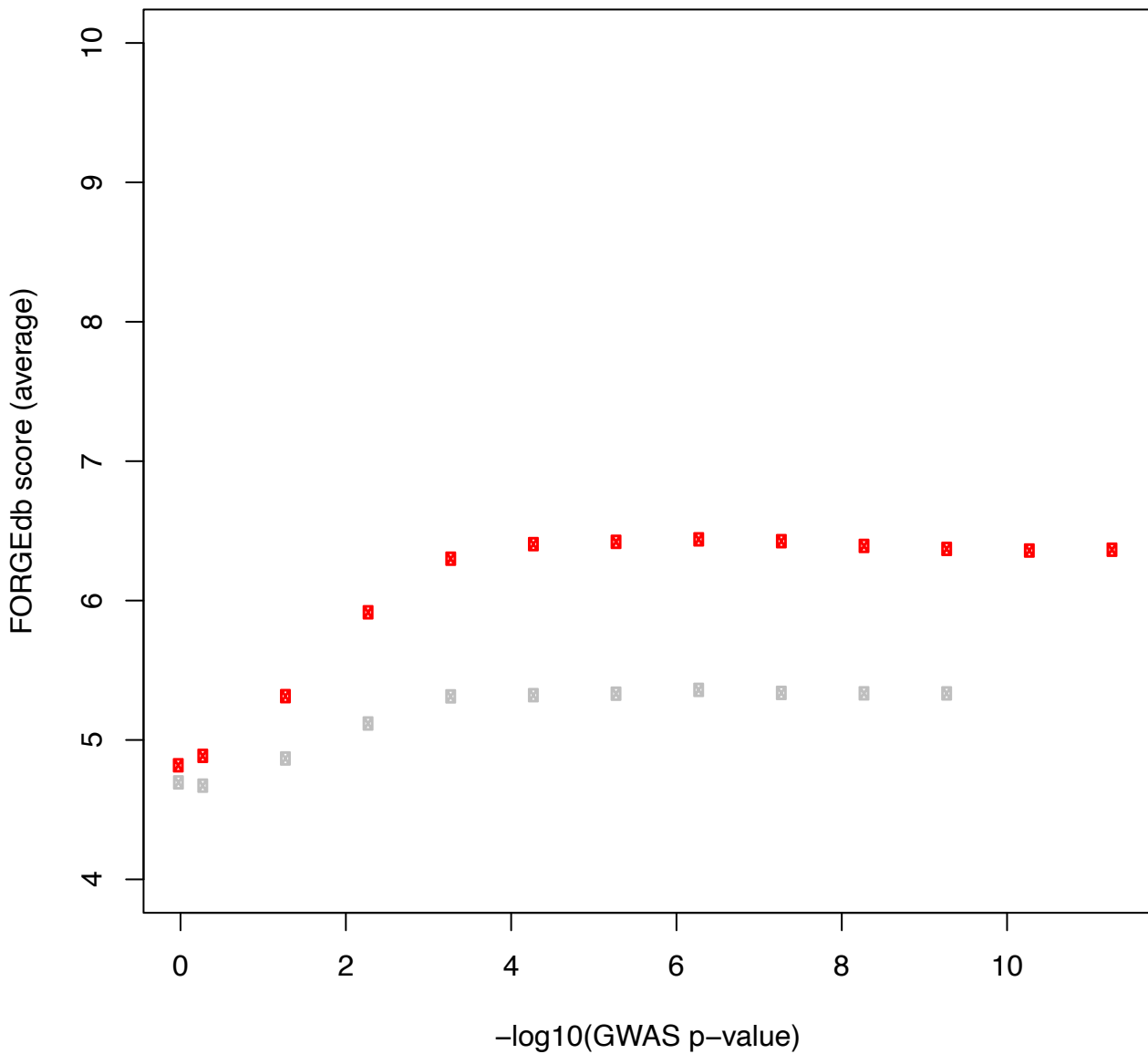

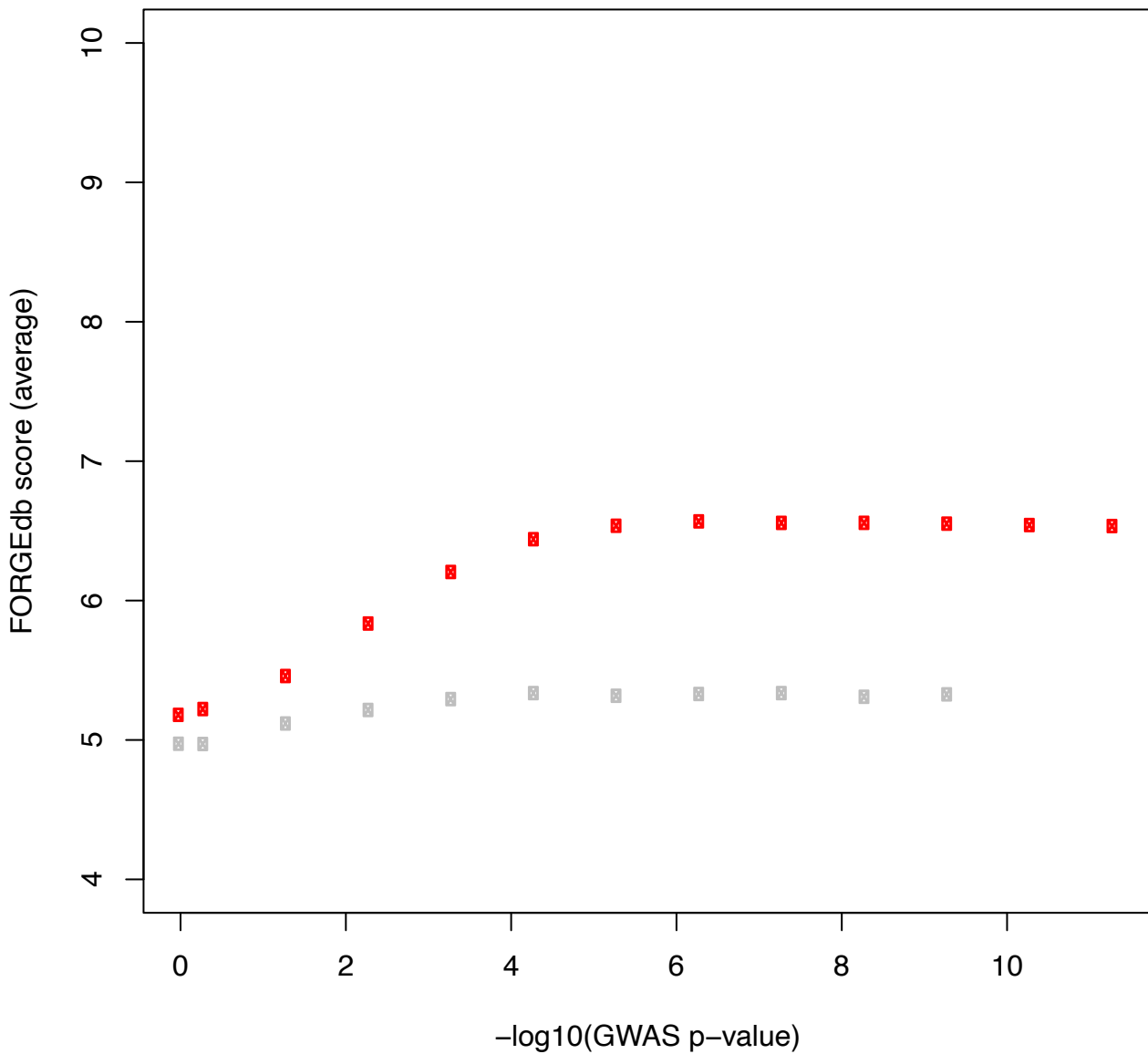

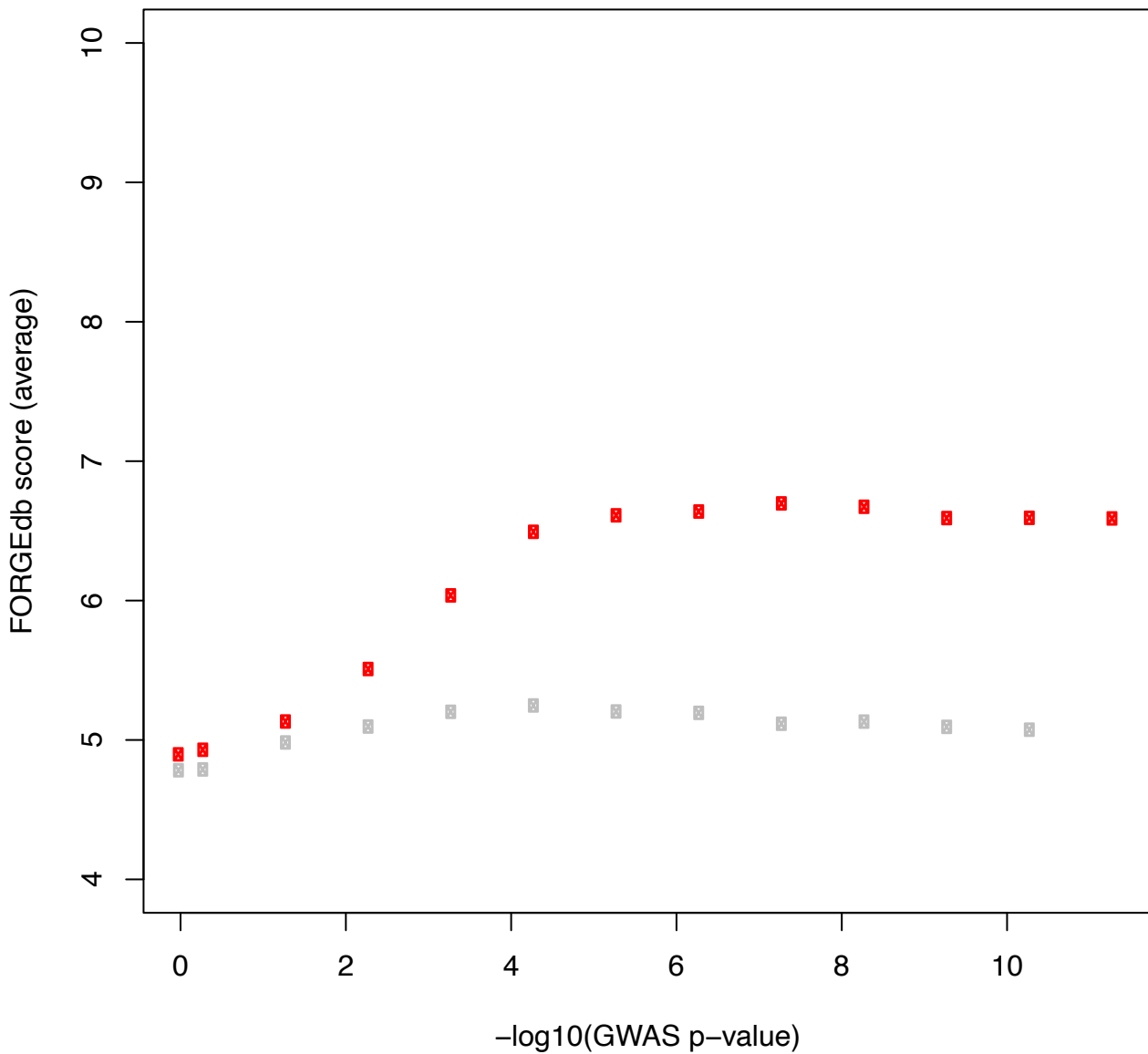

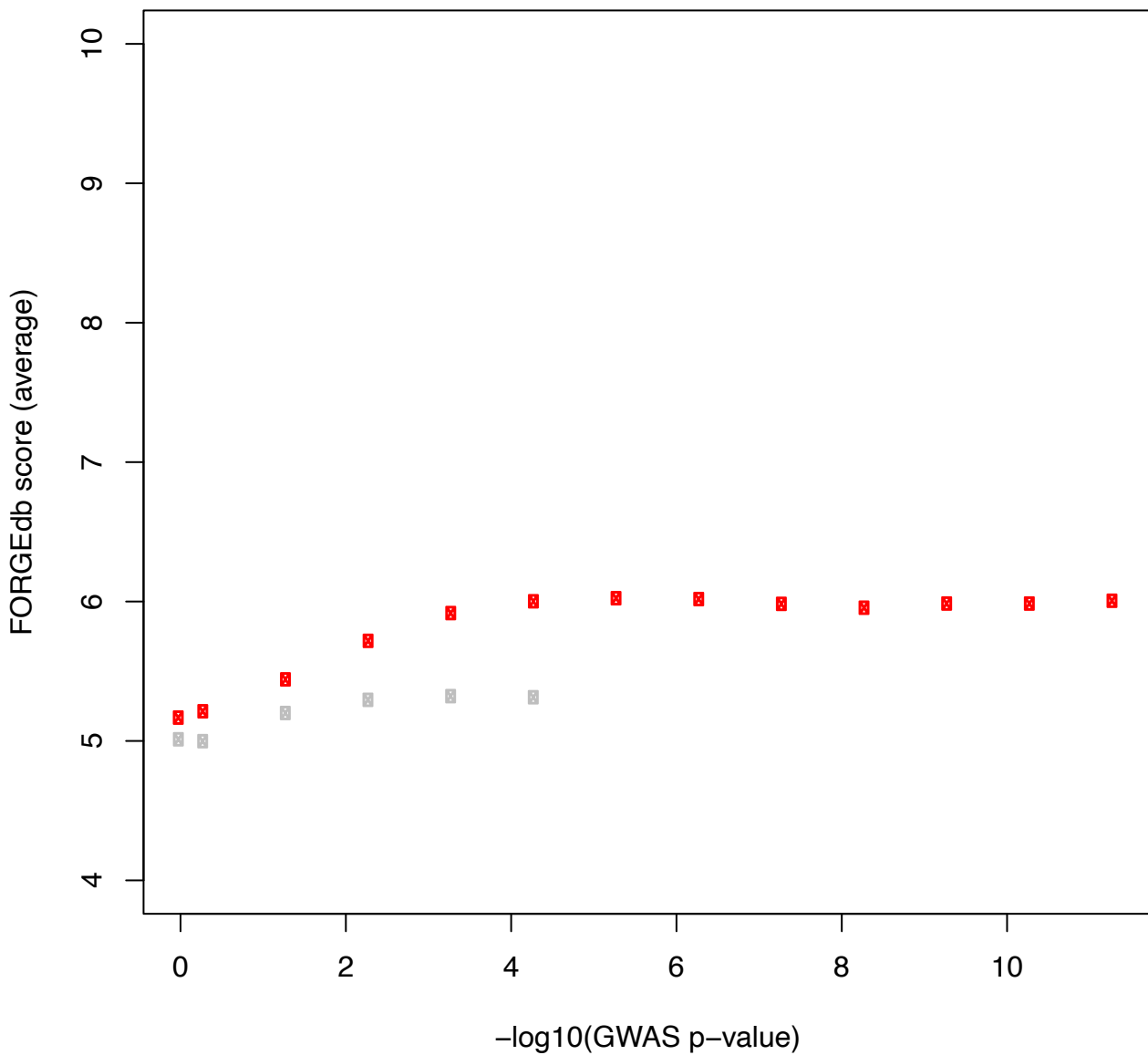

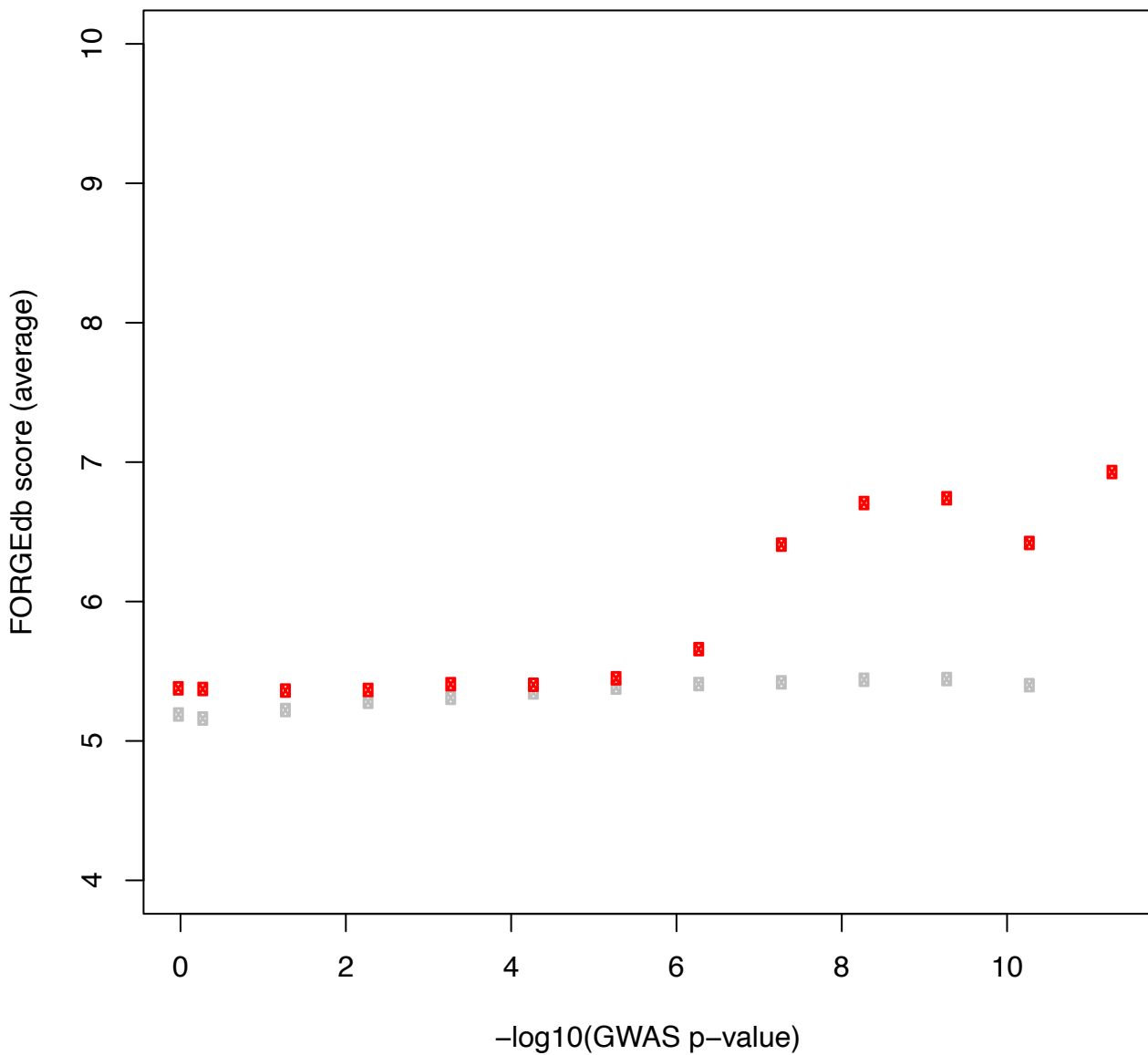

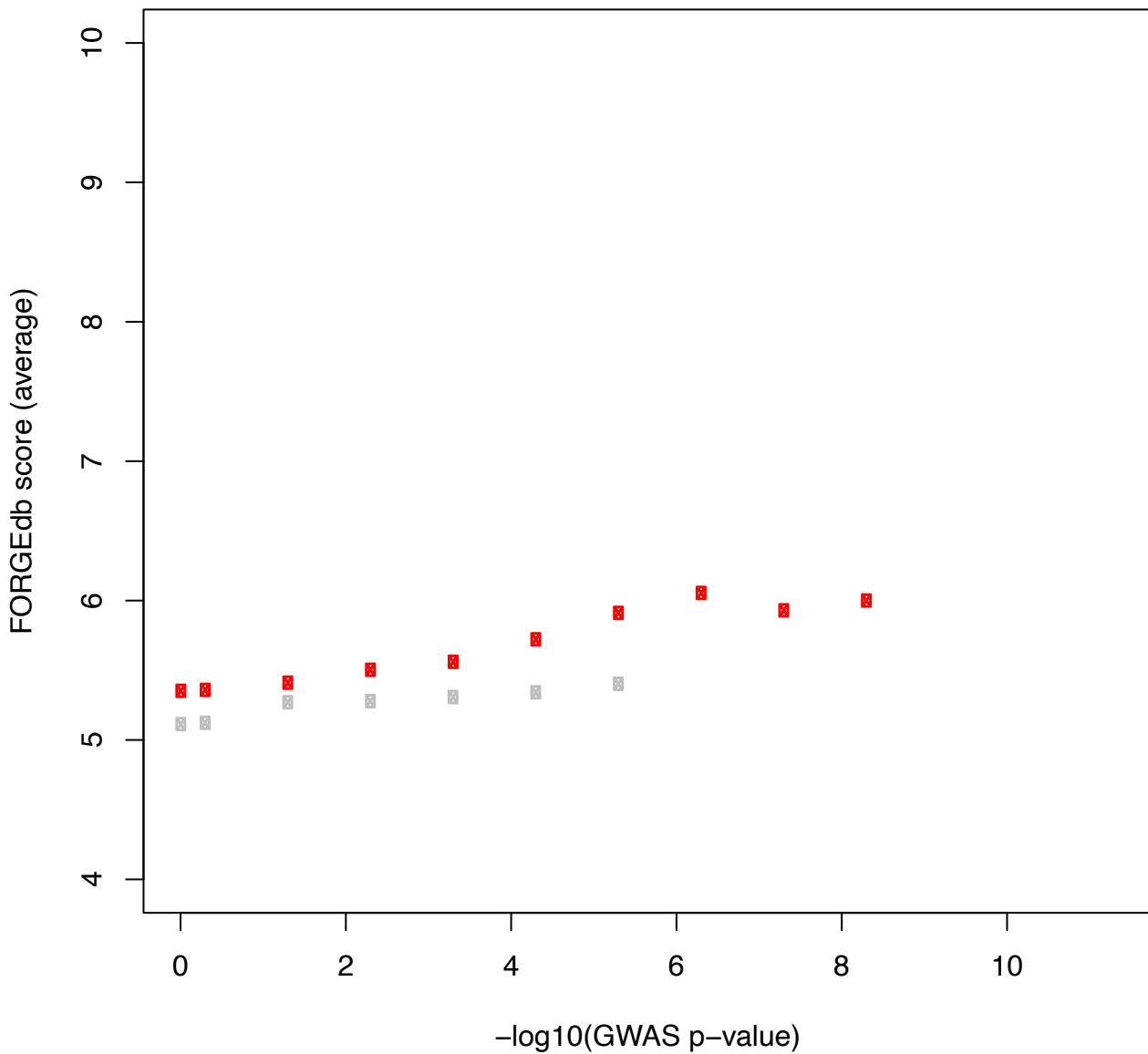

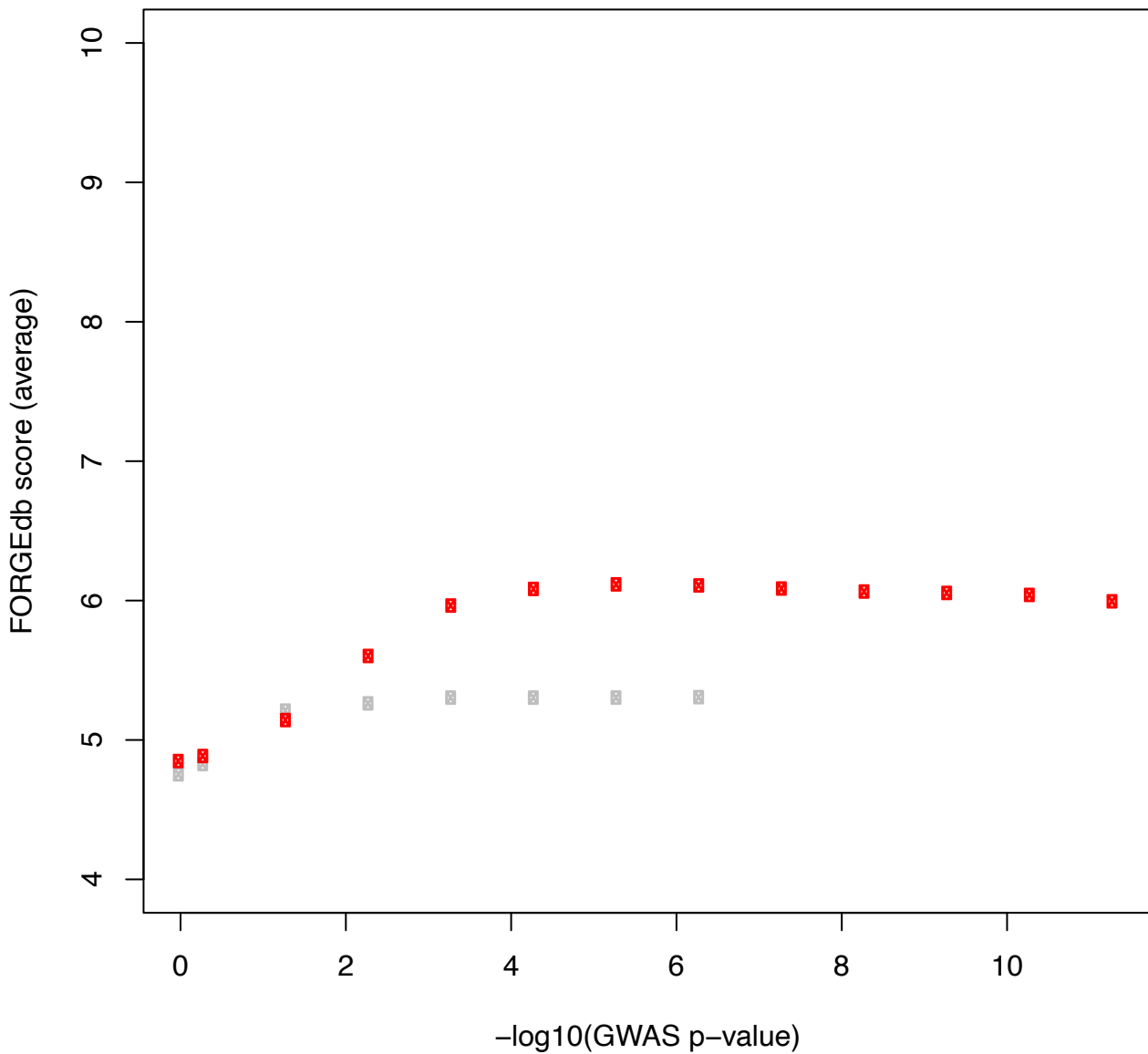

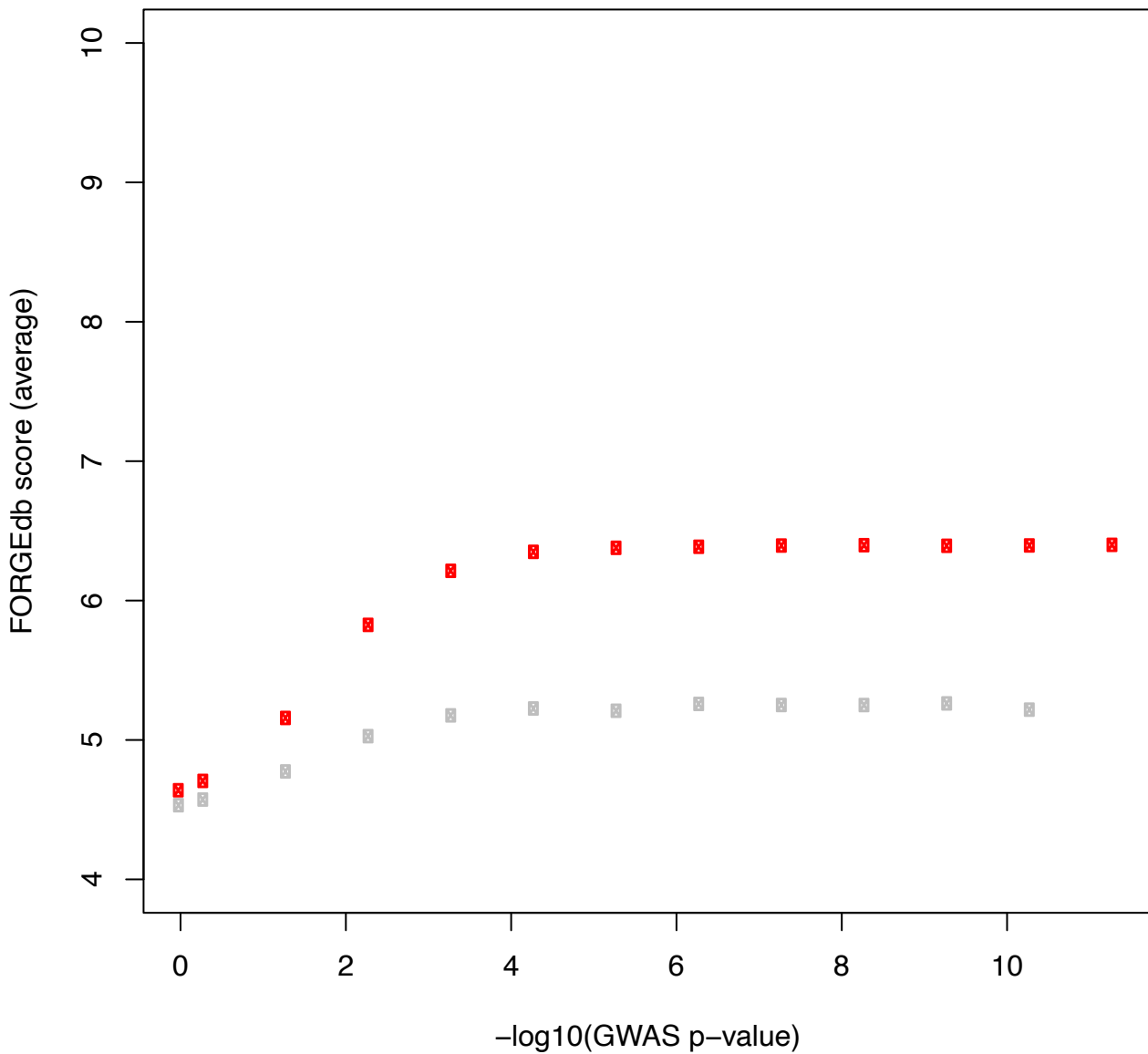

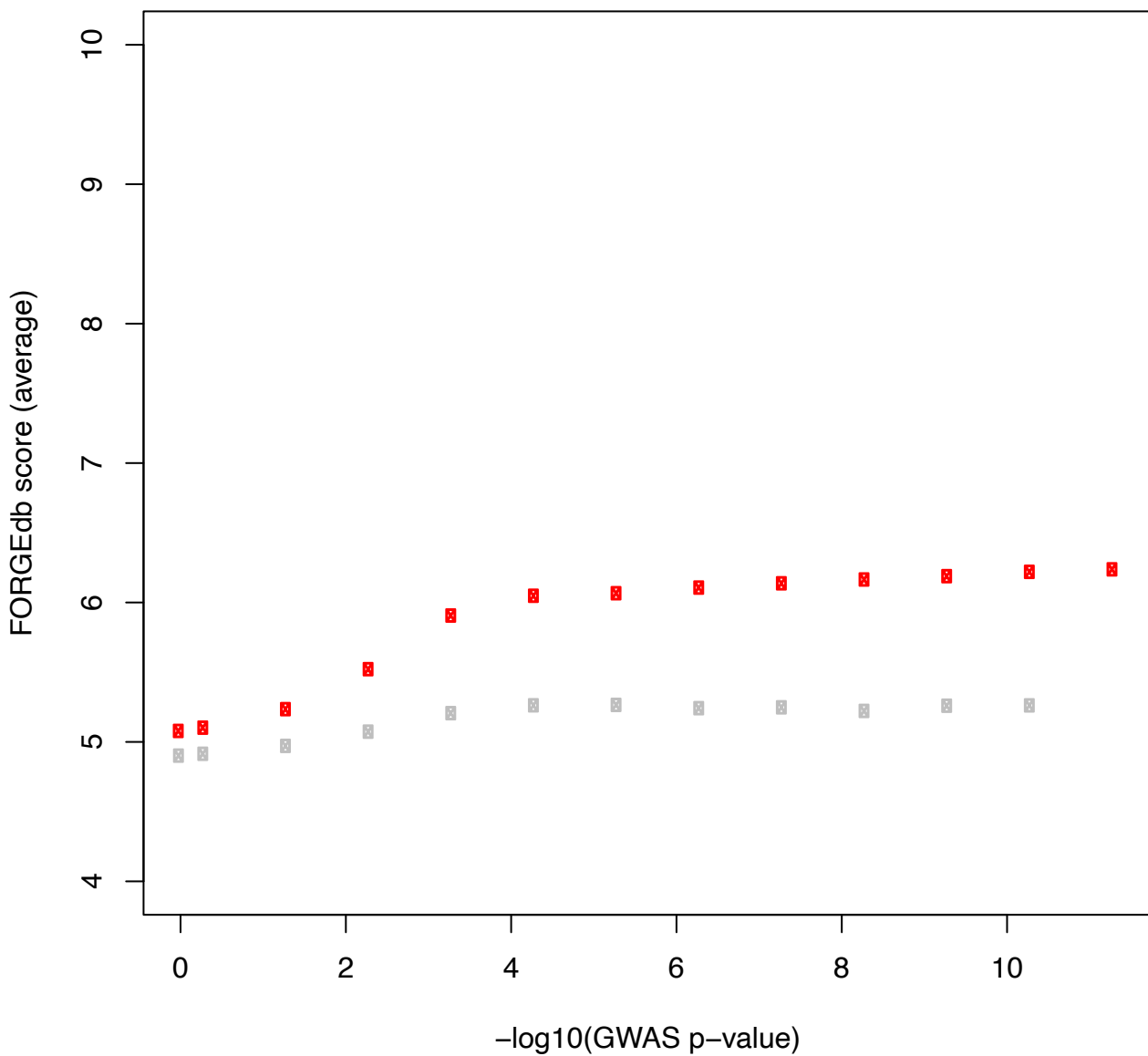

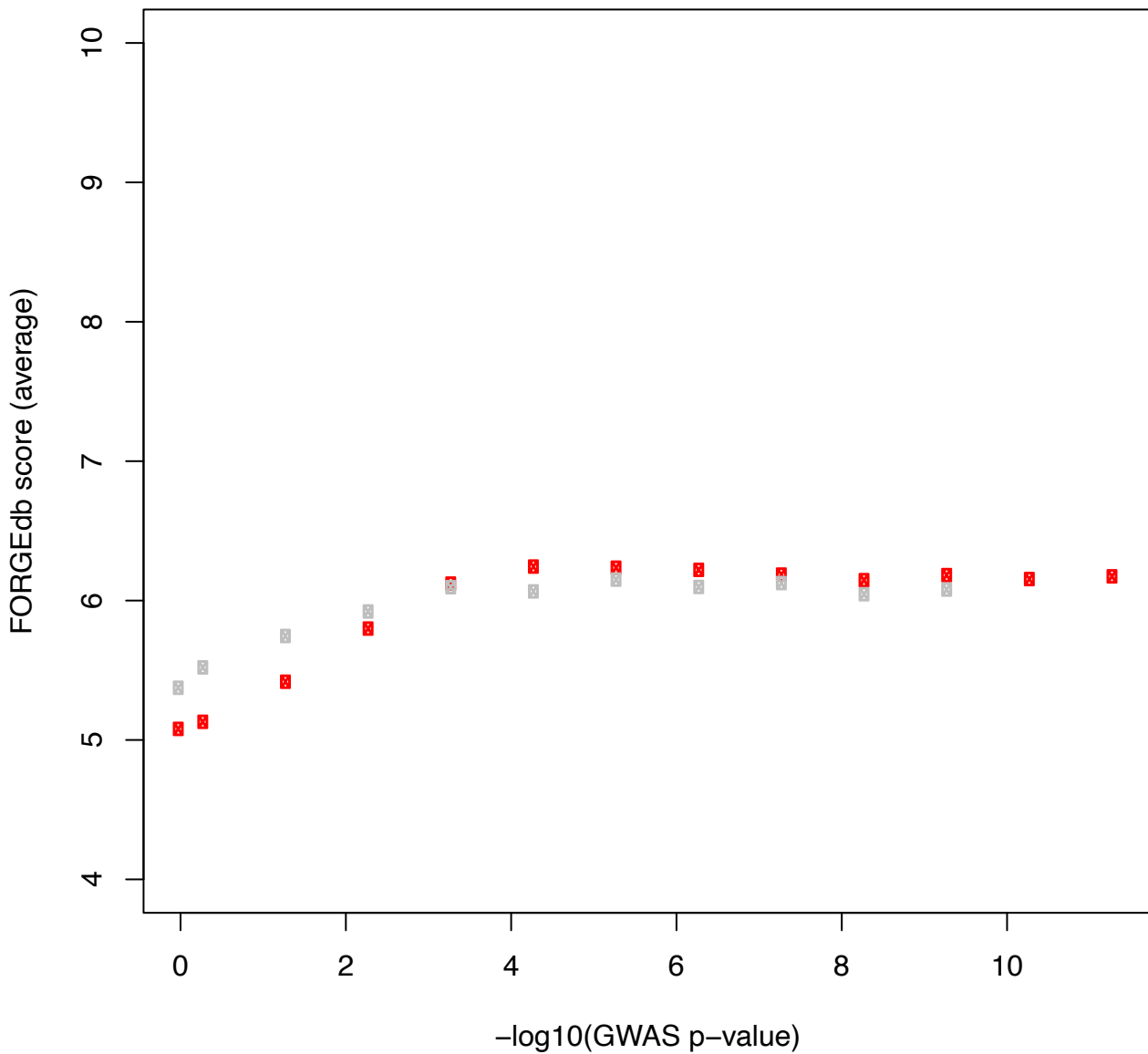

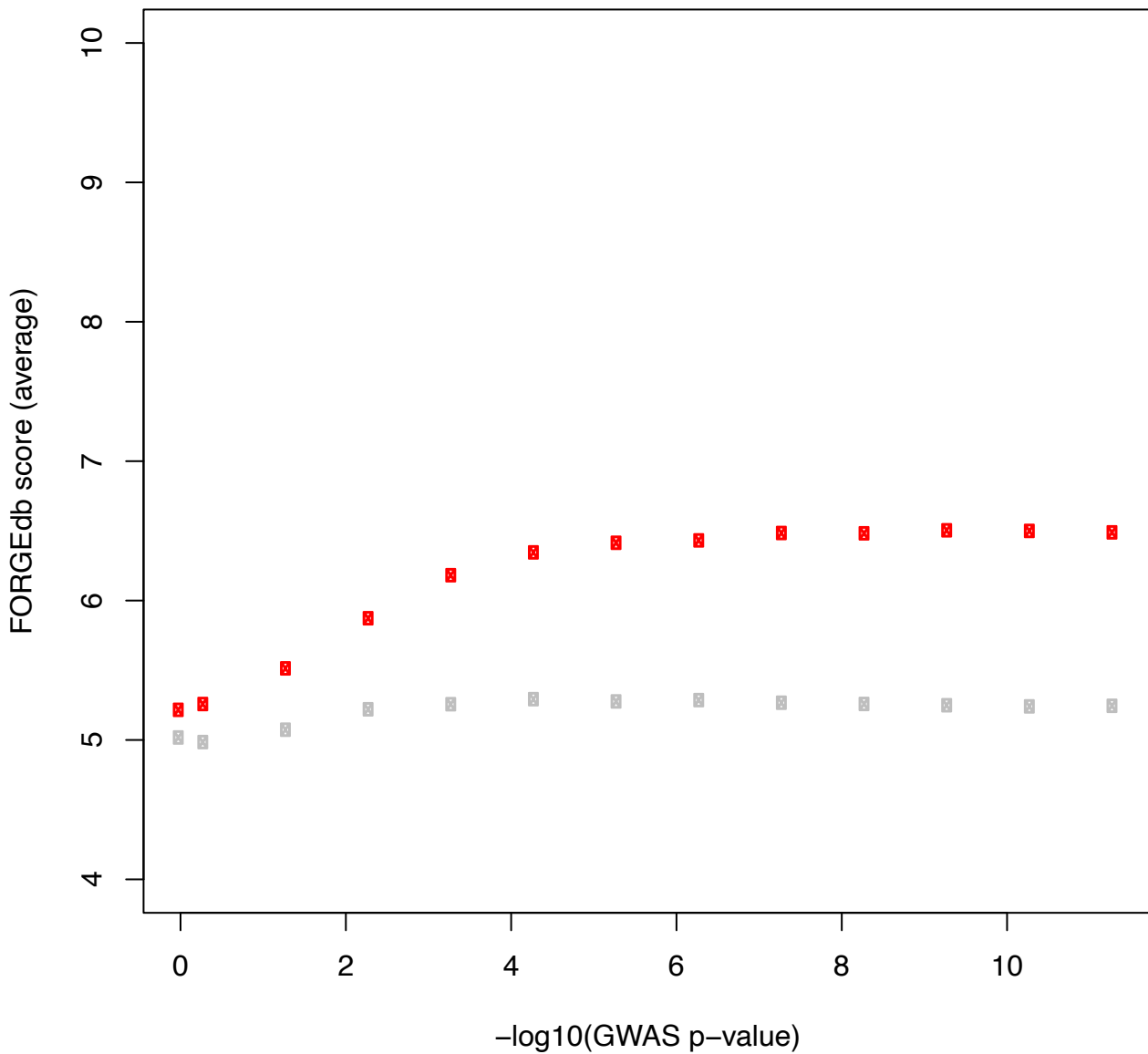

Supplement: Supplementary file 1 — Additional file 1. FORGEdb score (average, y-axis) versus GWAS -log10(p-value) (x-axis) across 30 GWAS, FORGE2 analysis. Each red point shows the FORGEdb score average across all GWAS SNPs at a each p-value cutoff. Each grey point shows the FORGEdb score average across background SNPs (FORGE2 linkage disequilibrium analysis, same minor allele frequency). FORGE2 SNP number requirements preclude background analysis for certain p-value thresholds in some of the GWAS. Order of panels: melanoma, monocyte cell count, diastolic blood pressure, systolic blood pressure, neutrophil cell count, eosinophil cell count, lymphocyte cell count, white blood cell count, basophil cell count, lung cancer, schizophrenia, estimated glomerular filtration rate, major depressive disorder, type 2 diabetes, body mass index, rheumatoid arthritis, height, waist-to-hip ratio, fasting insulin, red blood cell count, inflammatory bowel disease, Alzheimer’s disease, breast cancer, attention deficit hyperactivity disorder, autism, prostate cancer, LDL, hair color, colon cancer, and venous thrombosis. [file 13059_2023_3126_MOESM1_ESM.pdf]
