# Supplementary material for: FORGEdb: a tool for identifying candidate functional variants and uncovering target genes and mechanisms for complex diseases
Source: Genome Biol. 2024 Jan 2;25:3. doi: 10.1186/s13059-023-03126-1 (PMC10763681; doi:10.1186/s13059-023-03126-1)
Supplement: Supplementary file 3 — Additional file 3. Instructions for hosting FORGEdb on a static file server. [file 13059_2023_3126_MOESM3_ESM.docx]

**Additional file 3: Instructions for hosting FORGEdb on a static file server**

### [Hosting FORGEdb on a Static File Server](https://github.com/CBIIT/nci-webtools-dceg-forgedb#hosting-forgedb-on-a-static-file-server)

#### [Prerequisites](https://github.com/CBIIT/nci-webtools-dceg-forgedb#prerequisites)

Building

- git
- node.js

Hosting

- Any static file server. The example instructions use Apache.

#### [Instructions](https://github.com/CBIIT/nci-webtools-dceg-forgedb#instructions)

##### [Building and hosting the API](https://github.com/CBIIT/nci-webtools-dceg-forgedb#building-and-hosting-the-api)

Each dataset is available for download at the following urls:

| **Dataset** | **Version** | **URL** | **Notes** | |
| --- | --- | --- | --- | --- |
| abc | v1.0 | <https://forgedb.cancer.gov/api/abc/v1.0/abc.forgedb.csv.gz> | |  |
| cadd | v1.0 | <https://forgedb.cancer.gov/api/cadd/v1.0/cadd.forgedb.csv.gz> | |  |
| cato | v1.0 | <https://forgedb.cancer.gov/api/cato/v1.0/cato.forgedb.csv.gz> | |  |
| closest_gene | v1.0 | <https://forgedb.cancer.gov/api/closest_gene/v1.0/closest_gene.forgedb.csv.gz> | |  |
| eqtlgen | v1.0 | <https://forgedb.cancer.gov/api/eqtlgen/v1.0/eqtlgen.forgedb.csv.gz> | |  |
| forge2.blueprint | v1.0 | <https://forgedb.cancer.gov/api/forge2.blueprint/v1.0/forge2.blueprint.forgedb.csv.gz> | |  |
| forge2.encode | v1.0 | <https://forgedb.cancer.gov/api/forge2.encode/v1.0/forge2.encode.forgedb.csv.gz> | |  |
| forge2.erc | v1.0 | <https://forgedb.cancer.gov/api/forge2.erc/v1.0/forge2.erc.forgedb.csv.gz> | |  |
| forge2.erc2-chromatin15state-all | v1.0 | [https://forgedb.cancer.gov/api/forge2.erc2-chromatin15state-all/v1.0/forge2.erc2-chromatin15state-all.{0-9}.forgedb.csv.gz](https://forgedb.cancer.gov/api/forge2.erc2-chromatin15state-all/v1.0/forge2.erc2-chromatin15state-all.%7B0-9%7D.forgedb.csv.gz) | | {0-9} indicates this dataset is split into 10 parts |
| forge2.erc2-DHS | v1.0 | <https://forgedb.cancer.gov/api/forge2.erc2-DHS/v1.0/forge2.erc2-DHS.forgedb.csv.gz> | |  |
| forge2.erc2-H3-all | v1.0 | [https://forgedb.cancer.gov/api/forge2.erc2-H3-all/v1.0/forge2.erc2-H3-all.{0-9}.forgedb.csv.gz](https://forgedb.cancer.gov/api/forge2.erc2-H3-all/v1.0/forge2.erc2-H3-all.%7B0-9%7D.forgedb.csv.gz) | | {0-9} indicates this dataset is split into 10 parts |
| forge2.forge2tf | v1.0 | <https://forgedb.cancer.gov/api/forge2tf/v1.0/forge2tf.forgedb.csv.gz> | |  |
| gtex | v1.0 | <https://forgedb.cancer.gov/api/gtex/v1.0/gtex.forgedb.csv.gz> | |  |
| ENCODE4 CRISPR sgRNAs | v1.0 | <https://doi.org/10.5281/zenodo.10067458> | |  |
| zoonomia | v1.0 | <https://forgedb.cancer.gov/api/zoonomia/v1.0/zoonomia.forgedb.csv.gz> | |  |

1. Clone the repository: git clone https://github.com/CBIIT/nci-webtools-dceg-forgedb.git
2. Under your static file server's document root, create an api folder under which you wish to host the FORGEdb api.
3. Copy the contents of the client/public/api folder to the api folder you created in step 2.
4. Download source datasets from the table above to the appropriate folder. For example, place abc.forgedb.csv.gz under api/abc/v1.0/.
5. Navigate to the repository's database folder
6. Run npm install to install dependencies
7. For each dataset, execute the import.js script. For example: node import.js $DOCUMENT_ROOT/api/abc/v1.0/abc.forgedb.csv.gz

##### [Building and hosting the API Client (Website)](https://github.com/CBIIT/nci-webtools-dceg-forgedb#building-and-hosting-the-api-client-website)

1. Clone the repositrory: git clone https://github.com/CBIIT/nci-webtools-dceg-forgedb.git
2. Navigate to the client folder and run npm install && npm run build to generate the out folder. If your api is served under a subpath (eg: https://your_hostname/your_subpath/api/), specify the subpath as the NEXT_PUBLIC_BASE_PATH environment variable before building (eg: export NEXT_PUBLIC_BASE_PATH=/your_subpath).
3. Upload the out folder to your static file server's document root, ensuring that you do not overwrite the contents of the api folder.

### [Hosting FORGEdb on AWS S3 using Cloudfront](https://github.com/CBIIT/nci-webtools-dceg-forgedb#hosting-forgedb-on-aws-s3-using-cloudfront)

AWS S3/Cloudfront is a cost-effective, high-performance method of hosting FORGEdb. The recommended approach is to split the API and website code across two S3 Buckets so they can be managed independently.

1. Create or log in to an AWS Account
2. Create two S3 buckets (eg: ${org-name}-forgedb-api, ${org-name}-forgedb-website)
3. Create a Cloudfront distribution with an /api origin and a default origin. The /api origin should be served from ${$org-name}-forgedb-api, and the default origin should be served from ${org-name}-forgedb-website
4. Build the website code and push it to the $org-name-forgedb-website bucket
5. Copy the client/public/api folder to an api folder under the $org-name-forgedb-api bucket
6. Download the forgedb source files above
7. Upload each source file to the appropriate folder. For example, place abc.forgedb.csv.gz under api/abc/v1.0/.
8. Navigate to the repository's database folder
9. Run npm install to install dependencies
10. For each dataset, execute the import.js script. For example: node import.js s3://$org-name-forgedb-api/api/abc/v1.0/abc.forgedb.csv.gz. Ensure your environment has s3 credentials configured.
