# Supplementary material for: FORGEdb: a tool for identifying candidate functional variants and uncovering target genes and mechanisms for complex diseases
Source: Genome Biol. 2024 Jan 2;25:3. doi: 10.1186/s13059-023-03126-1 (PMC10763681; doi:10.1186/s13059-023-03126-1)
Supplement: Supplementary file 2 — Additional file 2. Review history. [file 13059_2023_3126_MOESM2_ESM.docx]

**1^st^ round**

**Reviewer 1**

This paper describes FORGEdb, a new web-based tool and associated database for annotating single-nucleotide variants in noncoding regions. FORGEdb defines a new scoring metric to identify important genetic variants/sites that are likely to be functional and makes these FORGEdb scores and related data available via a web interface. This seems like a useful innovation, as combining many different types of genomic data is often complex and challenging for researchers to manage on their own.

However, several points remain to be clarified and the paper is written in a very narrow way, focusing exclusively on FORGEdb's capabilities and not fully engaging with FORGEdb's place in the broader landscape. I'm also not sure about the future viability of FORGEdb because the website was down when I tried to use it and the FORGEdb dataset was not mirrored elsewhere.

Major comments

1. The authors describe a system for FORGEdb scores, that combines multiple lines of evidence to infer whether a given position. I had a few questions about this approach.

FORGEdb score is for prioritising variants, but many of these features mark regions. The authors should clarify which lines of evidence are based on the position of the variant and which are based on the residue. For example, is the presence of a TF motif determined based on the reference allele or the alternate allele? FORGEdb score is still clearly very useful if it only prioritises genetic positions, but the text says it prioritises "genetic variants for functional validation."

How were the FORGEdb weightings chosen? Were other weights tried? Many of these features are highly correlated, so did the authors think about a non-linear combination of evidence? Expanding on the way the FORGEdb scoring was set up would help future researchers who are taking on a similar task in their own areas.

2. The authors looked at MPRA data from Tewhey et al. (2016) but there are many other MPRA studies. How were these variants chosen? The authors could examine a different type of MPRA dataset like Kircher et al. (2019; PMID 31395865) that performed saturation mutagenesis to demonstrate the utility of their approach on an alternative data type with a finer scale. Is FORGEdb able to distinguish between genetic variants with similar positions? This will be an increasingly important question as detailed maps of individual regulatory elements continue to be generated.

3. Are there any annotations that are included in Haploreg and RegulomeDB that are not included in FORGEdb? These should be included for fairness/completeness.

4. FORGEdb does not appear to have a versioning scheme or plans for future updates articulated. Are the authors planning on adding additional datasets? Is there a way for users to submit or integrate their own data, in particular to recalculate FORGEdb scores based on additional experimental evidence? This should be clarified. If there's no ability for users to add their own data to the system, then the useful life of the project is likely to be very limited.

5. FORGEdb files are only available from the main website, which was down at the time of this writing. The authors should strongly consider mirroring the data required for generating the FORGEdb pages (if not the pages themselves) on a third party archival server such as Zenodo, FigShare or Dryad. This would also enable them to issue stable DOIs for individual data releases (see previous point).

6. The primary script required to set up FORGEdb (and several other required scripts) are not present on GitHub and I wasn't able to access the download from the main website. The tutorial appendix and the GitHub documentation are extremely specific to the authors' home environment and should be updated to use environment variables for directories, cluster head node, etc. Obviously an expert user would need to make their own changes based on their own HPC environment, but the documentation as written is basically a log of what the author did rather than a set of scripts that could be run elsewhere.

The documentation falls short of guiding the user to a the desired result of a local version of FORGEdb. For example, there's no index.pl like in the FORGE2 repository or any other instructions on what to do with the compressed files in the "done.htmls" directory.

Minor comments
1. The labels in Figure 2 are way too small to read without zooming into the PDF. The authors should think of another way to present these data that works on a printed page.

2. The LDlink URLs are outdated and the manuscript should be updated accordingly. The site displays the following message when they are accessed: "LDlink Domain URL Change Notice
The LDlink domain has been migrated from nci.nih.gov to nih.gov. Please use the new URL to access the website and make API calls. The original urls will be automatically redirected to the new urls for the time being until further notice."

3. Is there a Python interface for accessing the data either directly from FORGEdb or from LDlink? The authors should mention it if so.

4. The authors can delete the .DS_Store MacOS cache file from the repository.

**Reviewer 2**

The manuscript ForgeDB by Breeze et al provides a database of annotations and an integrated score for ~37 million variants. This work appears useful and the authors provide information on its international usage. I was priorly familiar with the existence of this database as further evidence of its nascent impact. However, there are a number of important concerns with the manuscript and database in its current form which I list below:
1) There is limited information on how the database will account for increasingly changing genome builds. In fact, there is little information in the manuscript on the current builds.
2) The manuscript is not sophisticated in its use of eQTL data. Due to linkage disequilibrium, power and tissue-specificity, an arbitrary cutoff is effectively meaningless. The authors should consider using instead a fine-mapping posterior probability for a variant as being causal from the eQTL data.
3) The manuscript mentions tissue-specificity as important, however does not touch on this issue and the impact on its scores. This is a major limitation.
4) Figure 2 is not convincing as many of the enrichments might actually just be representing the background of where GWAS SNPs are located. The authors should consider nearby SNPs with comparable allele frequencies.
5) Further the comparisons in Figure 2 are not informative as they are not in reference to any other approach or single underlying data type. For example, even the ABC and CADD papers compared to a baseline predictive model.
6) The emVar validation is unsurprising or convincing. In fact, the Tewhey paper shows enrichments of specific epigenetic features. This would be better if there was some evidence that the integrated ForgeDB score did better than a composite of known enriched annotations already. Otherwise, this is simply showing the same thing in a different way and gives no understanding of what the score is providing.
7) Recent major studies have expanded understanding of genome conservation (i.e. zoonomia project) further highlighting constrained regulatory regions. While the CADD score has conservation data within it, it is not now outdated by these new resources. I would expect the authors to include these new conservation tracks.
8) Figure 3a shows point estimates but there must be some variance here based on selecting groups of 1000 SNPs.
9) Minor: References 8 and 11 should be described exactly. It is poor style to say "other consortia" but reference only one consortium there while specifically naming the other two.

**Authors’ response**

Reviewer #1: This paper describes FORGEdb, a new web-based tool and associated database for annotating single-nucleotide variants in noncoding regions. FORGEdb defines a new scoring metric to identify important genetic variants/sites that are likely to be functional and makes these FORGEdb scores and related data available via a web interface. This seems like a useful innovation, as combining many different types of genomic data is often complex and challenging for researchers to manage on their own.

However, several points remain to be clarified and the paper is written in a very narrow way, focusing exclusively on FORGEdb's capabilities and not fully engaging with FORGEdb's place in the broader landscape. I'm also not sure about the future viability of FORGEdb because the website was down when I tried to use it and the FORGEdb dataset was not mirrored elsewhere.

**Response**: Thank you for your constructive feedback, and we agree that several points need further clarification to strengthen the manuscript. In response to your comment about the narrow scope of the manuscript. We acknowledge that the original manuscript may have overly focused on highlighting FORGEdb's capabilities without sufficiently exploring its place in the broader genomic landscape. To address this, we have expanded the Introduction and Discussion sections to provide a more comprehensive overview of existing databases and tools and discuss the potential advantages and limitations of FORGEdb in comparison to other resources. We have revised the discussion section of the paper as follows:

“FORGEdb exhibits several strengths and limitations. Although FORGEdb contains data on TF motifs and CATO scores for allele-specific DNase-seq-based TF binding, it does not have data on chromatin accessibility quantitative trait loci (caQTL), which are a similar dataset present in RegulomeDB. Additionally, even though FORGEdb includes recent conservation scores from the Zoonomia project, it does not include information on sequence constraint from SiPhy, which is present in Haploreg. Despite these limitations, FORGEdb remains a valuable resource for researchers seeking a comprehensive and integrated platform to annotate SNPs and interpret functional elements in the genome, particularly within the context of gene regulation and allele-specific effects.

FORGEdb has several strengths. Leveraging many different annotations, as well as its own SNP scoring system, FORGEdb facilitates a comprehensive analysis of variants and their regulatory context. It utilizes different types of DNase-seq and histone mark data to provide a deeper understanding of genomic regulatory landscapes. An additional distinctive feature of FORGEdb is its integration of 3D genomic data, specifically ABC Hi-C-based data, which permits the exploration of complex chromatin interactions. Furthermore, FORGEdb incorporates CADD scores, providing further information about the potential deleterious effects of variant alleles. CADD scores, along with CATO scores, and allele-specific association data from GTEx v8 and eQTLGen enable researchers to explore allele-specific effects in the context of genomic functionality. Neither ABC nor CADD scores nor CATO scores are available in RegulomeDB or Haploreg. In addition, FORGEdb scores correspond with functional significance based on MPRA data and appear to be more informative for potential functional significance than the probability scores provided in RegulomeDB.”

Regarding the inaccessibility of the FORGEdb website, we apologize for any inconvenience caused by the website being temporarily (~1.5 days) down during your attempt to access FORGEdb. In response to this concern, we have taken immediate action to enhance the platform's stability and have mirrored the FORGEdb dataset on at the National Cancer Institute (<https://cbiit.github.io/nci-webtools-dceg-forgedb/>) to mitigate such issues in the future. Please note that the website is not yet fully loaded with all 37 million variants but an example updated and tissue-searchable page can be found at <https://cbiit.github.io/nci-webtools-dceg-forgedb/explore/?rsid=rs12203592>. This second website will be fully loaded with 37 million variants within a few weeks.

Major comments

1. The authors describe a system for FORGEdb scores, that combines multiple lines of evidence to infer whether a given position. I had a few questions about this approach.

FORGEdb score is for prioritising variants, but many of these features mark regions. The authors should clarify which lines of evidence are based on the position of the variant and which are based on the residue. For example, is the presence of a TF motif determined based on the reference allele or the alternate allele? FORGEdb score is still clearly very useful if it only prioritises genetic positions, but the text says it prioritises "genetic variants for functional validation."

**Response**: Some of the lines of evidence underlying FORGEdb scores are allele- or variant-based and others are based on the position or regional overlap. FORGEdb provides a range of functional genomic annotations that can be categorized as either regional overlap or variant-level features. Among the regional overlap features included in FORGEdb are ABC data, TF motifs, DNase hotspots, and histone mark broadpeaks, which offer insight into genomic contexts. Variant-level features consist of GTEx and eQTLGen allele-specific association data, allele-specific TF binding data (CATO scores), Zoonomia PhyloP scores, and CADD scores. These annotations collectively contribute to a comprehensive understanding of allele-specific effects and regional genomic functionality within FORGEdb.

We have clarified this in the revised manuscript. This has been incorporated into the text as follows:

“FORGEdb provides a range of functional genomic annotations that can be categorized as based on positional overlap (e.g., the variant is located in a genomic region demarcated by the annotation) or variant-level features (e.g., allelic differences at the locus are associated with a particular feature). Among the regional overlap features, FORGEdb includes ABC data, TF motifs, DNase hotspots, and histone mark broadpeaks, offering insight into genomic context. Variant-level features, such as GTEx and QTLGen eQTL datasets, CATO scores, Zoonomia PhyloP scores and CADD scores provide allele-specific information. Collectively, these annotations in FORGEdb contribute to a comprehensive understanding of allele-specific effects and regional genomic context for individual SNPs.”

“…Specifically, FORGEdb annotates variants for **overlap** with DNase I hotspots, histone mark broadPeaks, and chromatin states across a wide range of cell and tissue types. Second, within these candidate regulatory regions, FORGEdb integrates SNPs with transcription factor (TF) binding data via: a) the **overlap** with TF motifs and b) SNP-specific Contextual Analysis of TF Occupancy (CATO) scores, which provide a complementary line of evidence for TF binding computed from **allele-specific** TF occupancy data measured by DNase I footprinting [15]. Third, FORGEdb links SNPs to target genes by providing: a) the **overlap** between SNPs and enhancer-to-promoter looping regions (or other looping regions) using Activity-By-Contact (ABC) data [16] and b) **allele-specific** expression quantitative trait locus (eQTL) annotations using large-scale data from GTEx [13] and eQTLGen [14]. In addition, FORGEdb includes annotations from datasets that aid interpretation of protein-coding changes. Specifically, it includes **allele-specific** CADD scores, which measure the deleteriousness of SNPs using experimental data and simulated mutations [17]. By amalgamating these datasets into a single resource, FORGEdb offers an expanded set of annotations and a more comprehensive evaluation of individual variants beyond those provided by other commonly used webtools (**Table 1**) [9] [10] [11].”

How were the FORGEdb weightings chosen? Were other weights tried? Many of these features are highly correlated, so did the authors think about a non-linear combination of evidence? Expanding on the way the FORGEdb scoring was set up would help future researchers who are taking on a similar task in their own areas.

**Response**: The weightings for the FORGEdb scores were created to capture a broad range of experimental evidence from multiple data sources without overweighting or emphasizing one line of evidence over others. Our goal was to develop scores that were accessible to a wide range of researchers, including experimental researchers, and we prioritized transparency and ease of interpretation in the scoring system. We utilized a combination of literature research and expert knowledge to identify the most relevant annotations and then conducted analyses to determine the most useful strategy and validate our approach.

For variant interpretation it is important to investigate different lines of experimental evidence to gain a comprehensive picture of potential biological mechanisms. For example, in the study of the *FTO* locus by Claussnitzer *et al.*, different lines of evidence (e.g. genome editing, enhancer data, RNA-seq, and 4C-seq) were used to converge on a mechanistic understanding of the variant, rs1421085. In a similar way, to prioritize variants at a larger scale it is also important to examine multiple different lines of experimental evidence and include datasets that have employed an agnostic approach and are not limited to a specific gene(s) or genetic region(s) or applied on a single cell type, which could introduce bias. In developing FORGEdb, we included datasets in regulatory genomics that: a) have widespread and high-quality data spanning different tissues and b) represent different experiments geared at understanding a specific genomic phenomenon. This led us to prioritize 5 key areas, each with a characteristic experiment or data type:

1) Evidence of chromatin accessibility (DNase-seq). Although we could have chosen ATAC-seq or FAIRE-seq to evaluate chromatin accessibility, we opted to choose DNase-seq due to availability of widespread, high-quality tissue-specific data and excellent mapping of enhancers/intergenic regions and deep sequenced libraries. We focused on wide regulatory regions, namely DNase I hotspots, which have shown to exhibit cell-specific enrichment for GWAS variants consistent with potential biological function (as shown by Dunham I, Kulesha E, Iotchkova V, Morganella S, Birney E. FORGE: A tool to discover cell specific enrichments of GWAS associated SNPs in regulatory regions. bioRxiv. 2014;013045. and Maurano MT, Humbert R, Rynes E, Thurman RE, Haugen E, Wang H, et al. Systematic localization of common disease-associated variation in regulatory DNA. Science. 2012;337:1190–5.).

2) Evidence of histone marks at a given region (ChIP-seq). We chose to use the Roadmap H3-all dataset to provide evidence of histone marks as it: a) includes many primary tissues samples as opposed to only cell line data, which has sometimes proved problematic when extrapolating results to *in vivo* phenomena; b) covers 5 histone marks (H3K4me1, H3K4me3, H3K36me3, H3K9me3, H3K27me3) that are each representative of major regulatory processes in the cell (Roadmap Epigenomics Consortium, Kundaje A, Meuleman W, Ernst J, Bilenky M, Yen A, et al. Integrative analysis of 111 reference human epigenomes. Nature. 2015;518:317–30.). Other histone marks were considered (e.g. H3K27ac), but they were not available for all tissue or cell types analysed by Roadmap and therefore not included. For FORGEdb, we thought that it was crucial that each mark be a separate informative experiment, but also be sufficiently available for wide set of tissues or cell types, so that the scores generated were consistent and not biased by tissue type.

3) Evidence of 3D genomics interactions (activity-by-contact or ABC). 3D genomic assays provide a crucial layer of regulatory information and a separate experiment from DNase-seq or ChIP-seq. Enhancer regions, which often contain regulatory variants that affect gene expression, typically loop to gene promoters in 3D. Although we could have used data from 4C-seq and Hi-C,  previous work has shown that ABC is superior for predicting: a) target genes and b) integrating 3D genomics data with GWAS data (Fulco CP, Nasser J, Jones TR, Munson G, Bergman DT, Subramanian V, et al. Activity-by-contact model of enhancer-promoter regulation from thousands of CRISPR perturbations. Nat Genet. 2019;51:1664–9.). There is also high-quality ABC data spanning many tissues.

4) Evidence of an association with gene expression (quantitative trait locus or eQTL). Many regulatory variants affect gene expression. Expression quantitative trait locus (eQTL) data can provide evidence for differential expression, another useful layer of evidence that a given variant may have regulatory function. In FORGEdb, we included eQTL data from GTEx, spanning multiple tissues, as well as data from eQTLGen, a consortium that analysed a large number blood samples, giving us a fairly comprehensive set of eQTL annotations (GTEx Consortium. Human genomics. The Genotype-Tissue Expression (GTEx) pilot analysis: multitissue gene regulation in humans. Science. 2015;348:648–60., Võsa U, Claringbould A, Westra H-J, Bonder MJ, Deelen P, Zeng B, et al. Large-scale cis- and trans-eQTL analyses identify thousands of genetic loci and polygenic scores that regulate blood gene expression. Nat Genet. 2021;53:1300–10.).

5) Evidence of transcription factor (TF) motifs and CATO. Many individual genomic variants are known or posited to affect transcription factor binding sites (TFBSs), which are hard to map and analyze via ChIP-seq given the wide range (~1600) of TFs in the human genome. We adopted a two-pronged approach incorporating: a) TF motifs (based on DNA sequence), which are available for a wide range of TFs through JASPAR, TRANSFAC, Taipale and Uniprobe databases, incorporated into FORGE2-TF (<https://analysistools.cancer.gov/forge2-tf/#/forge2-tf> and <https://forge2-tf.altiusinstitute.org/>), and b) CATO scores, a measure of local allelic effect on chromatin accessibility, distinct from larger accessible regions such as hotspots (Maurano MT, Haugen E, Sandstrom R, et al. Large-scale identification of sequence variants influencing human transcription factor occupancy in vivo. Nat Genet. 2015;47:1393–401.).

Using these 5 lines of evidence, we then sought to create weights that were unbiased, transparent, and readily interpretable. We considered several different weighting options, including non-linear combinations, but wanted to avoid overweighting one line of evidence over others or undervaluing other lines of evidence.  After considering different weighting approaches, we settled on a final score having a range of 0 to 10, which weights each line of evidence equally (e.g., each line of evidence has a maximum value of 2). Given that 3D genomics, gene expression, chromatin accessibility, histone mark categories and TFBSs are analyzed via different experimental approaches, a score of 2 for each avoids a situation where any individual experimental approach dominates over the rest. When we applied this scoring system to 37 million variants, we observed a normal distribution (**Figure 3b**). We further validated the scoring by testing it on MPRA data, showing that it corresponds with likely functional significance.

We acknowledge that there is room from improvement in the FORGEdb scoring system and that other weighting systems may provide better discriminatory power. In future versions, we plan to test machine learning approaches (e.g. random forest) to generate unsupervised weightings as these may yield further insights into the identification of regulatory variants. If we can identify a new weighting system that provides greater discriminatory power than our current score, we will implement it in upcoming versions of FORGEdb, with its appropriate version number.

We agree that providing a comprehensive explanation of the scoring methodology is essential to assist future researchers undertaking similar tasks in their respective areas. In response to your valuable feedback, we have expanded the "Methods" section of the manuscript to include an account of the weighting selection process, as well as paragraph in the main text describing the development and rationale for FORGEdb scores.

Below is the paragraph that was added to the main text:

“These five lines of experimental evidence were chosen to based on likelihood of providing evidence of biological function, availability of high quality data across multiple tissues, and offering a distinct and different line of experimental evidence. To prioritize variants at a larger scale for further functional studies, it is critical to examine multiple different lines of experimental evidence to gain a comprehensive picture of potential biological mechanisms. It is also important to include datasets that have employed an agnostic approach and are not targeted to a specific gene(s) or genetic region(s) or limited to a single tissue type, which could introduce bias.”

Below is the additional text added to the methods:

“For all variants, we generated a FORGEdb score to reflect the extent of experimental evidence supporting possible functional significance. The objective was to ensure that FORGEdb scores were accessible to a wide array of researchers while emphasizing transparency and interpretability. Weightings were built to encompass a broad spectrum of experimental evidence from diverse data sources and to limit bias toward any particular line of evidence.

In creating the FORGEdb scores, we focused on datasets covering major areas of regulatory genomics with high-quality data across multiple tissues and identified five key types of experimental evidence, each associated with a distinct experiment or dataset:

1. Chromatin accessibility. Evidence of chromatin accessibility, which is important for gene regulation, was assessed based on positional overlap with DNase I hotspots from the Roadmap Epigenomics consortium, ENCODE and BLUEPRINT, as analyzed in FORGE2 [5–8].
2. Histone marks. Evidence for positional overlap with histone marks was assessed using broadPeak ChIP-seq data from the consolidated Roadmap H3-all dataset, which covers the 5 main histone marks analyzed across the main tissue set (H3K4me1, H3K4me3, H3K36me3, H3K9me3, H3K27me3) [7].
3. Activity-by-contact (ABC) 3D genomics interactions. Evidence of ABC 3D genomics interactions, predictive of target gene looping, was assessed using positional overlap with ABC regions [16].
4. Differential gene expression. Evidence of allelic associations with gene expression were assessed using expression quantitative trait locus (eQTL) data from GTEx and eQTLGen [13,14].
5. Transcription factors. Evidence of potential alteration of transcription factor binding was assessed by positional overlap with transcription factor (TF) motifs from FORGE2-TF (<https://analysistools.cancer.gov/forge2-tf/#/forge2-tf> and <https://forge2-tf.altiusinstitute.org/>) and Contextual Analysis of TF Occupancy (CATO) scores [15], which provide a measure of allele-specific associations with chromatin accessibility for a wide array of TFs.

Equal weights (2 points each) were assigned to each line of evidence to prevent bias originating from any one approach. Resulting points were then added to provide a final FORGEdb score ranging from 0 to 10. When we applied this scoring system to 37 million variants, we observed a normal distribution (**Figure 3b**). We further validated the scoring by assessing it against MPRA data, affirming its alignment with functional significance.”

2. The authors looked at MPRA data from Tewhey et al. (2016) but there are many other MPRA studies. How were these variants chosen? The authors could examine a different type of MPRA dataset like Kircher et al. (2019; PMID 31395865) that performed saturation mutagenesis to demonstrate the utility of their approach on an alternative data type with a finer scale. Is FORGEdb able to distinguish between genetic variants with similar positions? This will be an increasingly important question as detailed maps of individual regulatory elements continue to be generated.

**Response:** We greatly appreciate the reviewer's suggestion regarding the selection of MPRA datasets and the potential utility of FORGEdb in distinguishing genetic variants with similar positions. We have now assessed saturation mutagenesis data for the finer scale analysis from Kircher et al. (2019; PMID 31395865).  Briefly, we downloaded hg38 data from <https://kircherlab.bihealth.org/satMutMPRA/> and evaluated FORGEdb scores across the dataset for variants at p<0.001. Our findings indicate that higher FORGEdb scores tend to perform effectively even when distinguishing variants located in close proximity from Kircher et al. (figure below). We have highlighted this in the revised manuscript as **Figure 3c**.

3. Are there any annotations that are included in Haploreg and RegulomeDB that are not included in FORGEdb? These should be included for fairness/completeness.

**Response**: Yes, RegulomeDB has chromatin accessibility quantitative trait loci (caQTLs), and Haploreg has data on sequence constraint from SiPhy cons. We have included these in the revised table 1. We have also revised our discussion to provide a more in depth discussion of how our tool compares with other available tools:

“FORGEdb exhibits several strengths and limitations. Although FORGEdb contains data on TF motifs and CATO scores for allele-specific DNase-seq-based TF binding, it does not have data on chromatin accessibility quantitative trait loci (caQTL), which are a similar dataset present in RegulomeDB. Additionally, even though FORGEdb includes recent conservation scores from the Zoonomia project, it does not include information on sequence constraint from SiPhy, which is present in Haploreg. Despite these limitations, FORGEdb remains a valuable resource for researchers seeking a comprehensive and integrated platform to annotate SNPs and interpret functional elements in the genome, particularly within the context of gene regulation and allele-specific effects.”

4. FORGEdb does not appear to have a versioning scheme or plans for future updates articulated. Are the authors planning on adding additional datasets? Is there a way for users to submit or integrate their own data, in particular to recalculate FORGEdb scores based on additional experimental evidence? This should be clarified. If there's no ability for users to add their own data to the system, then the useful life of the project is likely to be very limited.

**Response**: We have added a versioning scheme as part of an improved, modular FORGEdb implementation at <https://github.com/CBIIT/nci-webtools-dceg-forgedb/blob/forgedb_1.0.0/client/public/api/datasets.json>

5. FORGEdb files are only available from the main website, which was down at the time of this writing. The authors should strongly consider mirroring the data required for generating the FORGEdb pages (if not the pages themselves) on a third party archival server such as Zenodo, FigShare or Dryad. This would also enable them to issue stable DOIs for individual data releases (see previous point).

**Response**: We have created a new, modular version of FORGEdb at NCI. An example tissue-searchable page can be viewed at <https://cbiit.github.io/nci-webtools-dceg-forgedb/explore/?rsid=rs12203592>.  The full release of all SNPs will be available in a few weeks. We have also uploaded FORGEdb scores to Zenodo at <https://doi.org/10.5281/zenodo.8293408>.

6. The primary script required to set up FORGEdb (and several other required scripts) are not present on GitHub and I wasn't able to access the download from the main website. The tutorial appendix and the GitHub documentation are extremely specific to the authors' home environment and should be updated to use environment variables for directories, cluster head node, etc. Obviously an expert user would need to make their own changes based on their own HPC environment, but the documentation as written is basically a log of what the author did rather than a set of scripts that could be run elsewhere. The documentation falls short of guiding the user to a the desired result of a local version of FORGEdb. For example, there's no index.pl like in the FORGE2 repository or any other instructions on what to do with the compressed files in the "done.htmls" directory.

**Response**: We have created a completely new github repository using a more modular design at <https://github.com/CBIIT/nci-webtools-dceg-forgedb>. The new github repository now uses docker, allowing for much easier installation and usability. We have also built a completely public API, available from <https://cbiit.github.io/nci-webtools-dceg-forgedb/api-access/>.

Minor comments

1. The labels in Figure 2 are way too small to read without zooming into the PDF. The authors should think of another way to present these data that works on a printed page.

**Response:** This has been done. Figure 2 has been amended for readability, by collapsing repeated labels and increasing font size.

2. The LDlink URLs are outdated and the manuscript should be updated accordingly. The site displays the following message when they are accessed: "LDlink Domain URL Change Notice

The LDlink domain has been migrated from nci.nih.gov to nih.gov. Please use the new URL to access the website and make API calls. The original urls will be automatically redirected to the new urls for the time being until further notice."

**Response**: The URLs have been updated in the manuscript.

3. Is there a Python interface for accessing the data either directly from FORGEdb or from LDlink? The authors should mention it if so.

**Response**: We have provided several methods to access the data outside of the FORGEdb GUI webpage: via R (https://github.com/machiela-lab/LDlinkR), via the FORGEdb API (https://cbiit.github.io/nci-webtools-dceg-forgedb/api-access/, modular design, will be released in a few weeks), and via LDproxy, LDassoc and LDmatrix applications (https://ldlink.nih.gov). Of these, access using the FORGEdb API is likely the most relevant for python users. We will consider building python-specific applications in future releases.

4. The authors can delete the .DS_Store MacOS cache file from the repository.

**Response**: This has been done.

Reviewer #2: ===

The manuscript ForgeDB by Breeze et al provides a database of annotations and an integrated score for ~37 million variants. This work appears useful and the authors provide information on its international usage. I was priorly familiar with the existence of this database as further evidence of its nascent impact. However, there are a number of important concerns with the manuscript and database in its current form which I list below:

1)      There is limited information on how the database will account for increasingly changing genome builds. In fact, there is little information in the manuscript on the current builds.

**Response:** We are grateful for the reviewer's comment. FORGEdb is a SNP-based tool that uses rsIDs, which are robust to different genome builds, as its input (and for indexing). Due to this, FORGEdb does not use positional information to index each SNP, which therefore makes translating across genome builds much easier.

In addition, where relevant for annotation description, we have also included genome build information in table headers as part of an improved, modular design of FORGEdb (example updated and tissue-searchable page at <https://cbiit.github.io/nci-webtools-dceg-forgedb/explore/?rsid=rs12203592>).

2)      The manuscript is not sophisticated in its use of eQTL data. Due to linkage disequilibrium, power and tissue-specificity, an arbitrary cutoff is effectively meaningless. The authors should consider using instead a fine-mapping posterior probability for a variant as being causal from the eQTL data.

**Response:** We are grateful for the reviewer's insightful comment regarding the use of eQTL data in our manuscript. The reviewer is correct that it would be more informative to do a colocalization analysis and determine the posterior probability of a variant being causal. However, this would require the user to upload their summary statistics for the region and our program to run a colocalization analysis. Regarding summary statistics and integration with FORGEdb, there are two aspects:

a) direct webtool integration: At this point, direct webtool integration of summary statistics is outside the scope of our tool, which focuses on individual positions/rsIDs and single variant-level annotations, which by necessity involves not being able to incorporate GWAS summary statistics across multiple variants into the analysis. However, we are excited about building such a method in the future, in which a user could upload their summary statistics and colocalization analyses would be performed. We would welcome advice on this future method, we would welcome contact at [c.breeze@ucl.ac.uk](mailto:c.breeze@ucl.ac.uk).

b) integration of FORGEdb scores with GWAS summary statistics: as a computational score for individual variants scaled between 0 and 10, it is possible to integrate FORGEdb scores (downloadable from Zenodo at <https://doi.org/10.5281/zenodo.8293408>) with summary statistics via computational models, and we are excited about the integration/analysis of summary statistics and FORGEdb scores.

We have added a section in the text regarding both of these points:

Integration with summary statistics

“Although FORGEdb does include blood cis-eQTL data from a large consortium, eQTLGen, offering additional information beyond GTEx, the FORGEdb webtool does not currently conduct colocalization analyses and thus does not compute the posterior probability of a variant affecting gene expression for a given GWAS. Regarding applications for summary statistics, we recommend modelling analysis in R using computed FORGEdb scores across over 37 million variants, which are scaled between 0 and 10 and are available for download at Zenodo at <https://doi.org/10.5281/zenodo.8293408>, to facilitate integration and joint analysis of summary statistics and FORGEdb scores.”

3)      The manuscript mentions tissue-specificity as important, however does not touch on this issue and the impact on its scores. This is a major limitation.

**Response:** This is an excellent point. In our updated tool, we have incorporated strategies to address tissue specificity, recognizing its importance. We now include a tissue-specific search and scoring as part of an improved, modular design of FORGEdb (example updated and tissue-searchable page at <https://cbiit.github.io/nci-webtools-dceg-forgedb/explore/?rsid=rs12203592>). Specifically, when a tissue is searched on the new FORGEdb page, result tables are filtered for the given tissue and the score is recalculated from available tables.

4)      Figure 2 is not convincing as many of the enrichments might actually just be representing the background of where GWAS SNPs are located. The authors should consider nearby SNPs with comparable allele frequencies. Further the comparisons in Figure 2 are not informative as they are not in reference to any other approach or single underlying data type. For example, even the ABC and CADD papers compared to a baseline predictive model.

**Response:** We would like to thank the reviewer for their insightful feedback on Figure 2 of our manuscript. We understand the importance of ensuring the robustness and specificity of our enrichment analysis. In response to the concerns raised, we have conducted two additional analyses that address the potential confounding factors associated with the observed enrichments.

1. Minor Allele Frequency (MAF) Binning Analysis: To address the concern that the observed enrichments might be influenced by the background distribution of GWAS SNPs, we performed an analysis where we binned the SNPs based on their minor allele frequencies (MAF). By comparing enrichments in different MAF bins to the background distribution of SNPs within the same MAF range, we aimed to disentangle the potential influence of allele frequencies on the observed enrichments (updated **Figure 2**). This analysis supports the notion that the observed enrichments are not driven by the MAF distribution of GWAS SNPs.

2. MAF Background SNPs with LD Blocks in FORGE2: To further validate the enrichment patterns, we conducted an analysis using background SNPs selected from the general population using MAF bins identified in the FORGE2 database as well as FORGE2 LD (linkage disequilibrium, r2>0.8) block information. Results from this analysis are shown in **Supplementary File 1**, and also support the notion that the observed enrichments are not driven by the background of where the GWAS SNPs are located.

We have added the following to the main text:

To assess the potential utility of FORGEdb scores in large-scale analysis of GWAS data, we obtained summary statistics from published studies of 30 traits/diseases (**Methods**) [2,19–41] and evaluated the correlation between FORGEdb scores and the ranking of SNPs by association p-value in each GWAS. Specifically, we binned the SNPs according to their association -log10 p-value and estimated the mean FORGEdb score for each bin. Results revealed a significant positive correlation between mean FORGEdb score and ranked SNP bins across all 30 phenotypes, with more significant p-values corresponding to higher FORGEdb scores (**Figure 2 and Supplementary File 1**, median correlation=0.845, range 0.55 to 0.98). As fine-mapping studies can identify sets of variants more likely to be functional, we compared FORGEdb scores for variants from statistically-derived 95% credible sets with reported top SNPs from the same published study [42]. We discovered a significant overrepresentation of higher FORGEdb scores in the 95% credible sets (t-test p-value=0.002). These findings demonstrate that FORGEdb scores correlate with variants identified from GWAS and may be useful for prioritizing SNPs across a wide range of human traits and diseases, from common traits such as brown hair color and height to complex diseases like schizophrenia and lung cancer.

We have also added supplementary File 1

Supplementary File 1 legend:

**Supplementary File 1:** **FORGEdb score (average, y-axis) versus GWAS -log10(p-value) (x-axis) across 30 GWAS, FORGE2 analysis.** Each red point shows the FORGEdb score average across all GWAS SNPs at a each p-value cutoff. Each grey point shows the FORGEdb score average across background SNPs (FORGE2 linkage disequilibrium analysis, same minor allele frequency). FORGE2 SNP number requirements preclude background analysis for certain p-value thresholds in some of the GWAS. Order of panels: melanoma, monocyte cell count, diastolic blood pressure, systolic blood pressure, neutrophil cell count, eosinophil cell count, lymphocyte cell count, white blood cell count, basophil cell count, lung cancer, schizophrenia, estimated glomerular filtration rate, major depressive disorder, type 2 diabetes, body mass index, rheumatoid arthritis, height, waist to hip ratio, fasting insulin, red blood cell count, inflammatory bowel disease, Alzheimer’s disease, breast cancer, attention deficit hyperactivity disorder, autism, prostate cancer, LDL, hair colour, colon cancer, and venous thrombosis.

Updated Figure 2

We have therefore incorporated these findings, along with the suggested comparison to a baseline predictive model, into the revised manuscript to enhance the clarity and rigor of our study.

6)      The emVar validation is unsurprising or convincing. In fact, the Tewhey paper shows enrichments of specific epigenetic features. This would be better if there was some evidence that the integrated ForgeDB score did better than a composite of known enriched annotations already. Otherwise, this is simply showing the same thing in a different way and gives no understanding of what the score is providing.

**Response:** For the emVar paper, different regulatory genomic features were used to select candidate MPRA variants, including a) eQTL data from lymphoblastoid cell lines (“eQTLs from the Geuvadis RNA-seq dataset of lymphoblastoid cell lines (LCLs) from individuals of European (EUR) and West African (YRI) ancestry”) and b) LD information for variants proximal to these eQTLs and c) GWAS information for a set of loci which were analyzed more intensively. Thus, a compendium of eQTL, LD and GWAS information was used as a composite to prioritize a given set of genomic variants.

FORGEdb scores are also a composite of known enriched annotations, and, importantly,

validated emVars exhibited significantly higher FORGEdb scores than 39,487 candidate MPRA variants from the same study (paired t-test p-value=0.004), suggesting that FORGEdb scores add further information not present in the composite set of regulatory genomic features applied in the variant prioritization methods used by Tewhey *et al*. In short, emVars were chosen/prioritized based on a composite of different known enriched features. However, our FORGEdb score enrichments for validated emVars show that our score can potentially distinguish these more than the composite of different known enriched features that the methods used by Tewhey *et al*.

In addition, and to provide further information we performed additional comparisons with data from Kircher et al. (2019; PMID 31395865), as well as comparisons of FORGEdb scores with scores from RegulomeDB across Tewhey *et al*. and Kircher *et al*. datasets (updated Figure 3).

7)      Recent major studies have expanded understanding of genome conservation (i.e. zoonomia project) further highlighting constrained regulatory regions. While the CADD score has conservation data within it, it is not now outdated by these new resources. I would expect the authors to include these new conservation tracks.

**Response:** We thank the reviewer for this insightful comment. We have added genome conservation data from the Zoonomia project as a new table in our tool and updated our manuscript and webpage accordingly, as well as table 1 comparing FORGEdb to RegulomeDB and HaploReg.

Updated text:

To address this issue and provide researchers with a state-of-the-art web tool for variant annotation that includes these updated resources, we developed FORGEdb (<https://forge2.altiusinstitute.org/files/forgedb.html>, **Table 1**). FORGEdb incorporates a range of datasets covering three broad areas relating to gene regulation: regulatory regions, transcription factor (TF) binding, and target genes. First, using genome-wide epigenomic track data from ENCODE [6], Roadmap Epigenomics [7], and BLUEPRINT [8] consortia, FORGEdb links SNPs with data for candidate regulatory regions (e.g., enhancers or promoters). Specifically, FORGEdb annotates variants for overlap with DNase I hotspots, histone mark broadPeaks, and chromatin states across a wide range of cell and tissue types. Second, within these candidate regulatory regions, FORGEdb integrates SNPs with transcription factor (TF) binding data via: a) the overlap with TF motifs and b) SNP-specific Contextual Analysis of TF Occupancy (CATO) scores, which provide a complementary line of evidence for TF binding computed from allele-specific TF occupancy data measured by DNase I footprinting [15]. Third, FORGEdb links SNPs to target genes by providing: a) the overlap between SNPs and enhancer-to-promoter looping regions (or other looping regions) using Activity-By-Contact (ABC) data [16] and b) allele-specific expression quantitative trait locus (eQTL) annotations using large-scale data from GTEx [13] and eQTLGen [14]. In addition, FORGEdb includes annotations from datasets that aid interpretation of protein-coding changes. Specifically, it includes allele-specific CADD scores, which measure the deleteriousness of SNPs using experimental data and simulated mutations [17]. Moreover, FORGEdb includes the latest sequence conservation data from the Zoonomia project [18]. By amalgamating these datasets into a single resource, FORGEdb offers an expanded set of annotations and a more comprehensive evaluation of individual variants beyond those provided by other commonly used webtools (**Table 1**) [9] [10] [11].

8)      Figure 3a shows point estimates but there must be some variance here based on selecting groups of 1000 SNPs.

**Response:** In figure 3, “number of emVars per 1000 SNPs” is indicated, as a divisor (a bit like number of deaths per 100,000 individuals). It is hard to add error here, since it is a divisor for the full quantity. We apologize for the confusion, as this is not based on a randomized selection with groups of 1000 SNPs, but rather is a divisor. This has been clarified in the text.

9)      Minor: References 8 and 11 should be described exactly. It is poor style to say "other consortia" but reference only one consortium there while specifically naming the other two.

**Response:** This has been done. Reference 11 points to a review on >9 webtools and methods for integrating variants and epigenomic data. Reference 8 points to the BLUEPRINT consortium, this has been clarified in the text.

**2^nd^ round**

**Reviewer 1**

I would like to thank the authors for their thoughtful response to the initial round of review. They have done a substantial amount of work in response to the reviewer comments, including making extensive improvements to the software and associated documentation.

My major concerns with the manuscript text have been addressed, but I have a few minor comments focused on the new software.

1. The new datasets.json file includes a "versions" field that refers to the release of FORGEdb. However, this is quite confusing as many of these resources (e.g. CADD, GTEx) have their own version numbers. The current “versions” field should be changed to something like “forgedb_versions” for clarity.

The datasets.json and associated API endpoint should also support optional metadata for external resources, such as their actual version number (e.g. CADD v1.6 and GTEx v8 as described in manuscript Table 1), external website link, and publication DOI link. This will be helpful for users and promote reproducibility.

2. The dataset representations in the API are inconsistent and sometimes confusing, likely because this reflects the source data formats. For example, the closestGene information includes fields “chr” “start” and “end” for the rsID and “chr2” “start2” and “end2” for the gene. These field names are not descriptive, and important information (the strand for the gene) is missing.

CADD returns a “chromosome” field with an integer, in contrast to closestGene using a “chr” field with a “chrN” formatted string, and a single position for the rsID rather than a single-base-length start and end interval. GTEx has yet another set of conventions.

Standardizing features like genomic coordinates across the API would help users who want to consume the data and work with it themselves. However, this is quite complex and potentially fraught since FORGEdb would have to transform all the input data and would then be serving a version of established datasets that doesn’t match the original format. I do not think that this is needed for this version of FORGEdb, but it’s something that the authors can consider if they think it would be a useful extension of this work.

3. The new modular software and documentation are much improved. However, while the response mentions Docker and dockerfiles are present in the GitHub repository, there’s no instructions in the README for how to install the software using Docker and Docker is not mentioned as a dependency. This seems like a relatively minor oversight that could be corrected by adding a “Hosting FORGEdb with Docker” section to the existing README instructions. Retaining the instructions that don’t use Docker is valuable for users in the many institutional environments where Docker is not available.

4. The instructions state that “Any static file server” can be used for hosting. While I’m sure this is correct, it would be nice if the authors provided a recommendation and some accompanying basic instructions so that researchers with minimal experience can get something set up locally without needing to choose from the many static servers that are available.

**Reviewer 2**

The authors have sufficiently addressed my concerns

**Authors’ response**

Reviewer 1

1. The new datasets.json file includes a "versions" field that refers to the release of FORGEdb. However, this is quite confusing as many of these resources (e.g. CADD,

GTEx) have their own version numbers. The current “versions” field should be changed

to something like “forgedb_versions” for clarity. The datasets.json and associated API

endpoint should also support optional metadata for external resources, such as their

actual version number (e.g. CADD v1.6 and GTEx v8 as described in manuscript Table

1), external website link, and publication DOI link. This will be helpful for users and

promote reproducibility.

Response: this has been done (see https://raw.githubusercontent.com/CBIIT/nci-

webtools-dceg-

forgedb/26461e40b2a89951bbf79f123e5a11ad14d150c6/client/public/api/datasets.jso

n). We include a "metadata" field for each dataset which includes original resource

information.

2. The dataset representations in the API are inconsistent and sometimes confusing,

likely because this reflects the source data formats. For example, the closestGene

information includes fields “chr” “start” and “end” for the rsID and “chr2” “start2” and

“end2” for the gene. These field names are not descriptive, and important information

(the strand for the gene) is missing. CADD returns a “chromosome” field with an

integer, in contrast to closestGene using a “chr” field with a “chrN” formatted string, and

a single position for the rsID rather than a single-base-length start and end interval.

GTEx has yet another set of conventions. Standardizing features like genomic

coordinates across the API would help users who want to consume the data and work

with it themselves. However, this is quite complex and potentially fraught since

FORGEdb would have to transform all the input data and would then be serving a

version of established datasets that doesn’t match the original format. I do not think

that this is needed for this version of FORGEdb, but it’s something that the authors can

consider if they think it would be a useful extension of this work.

Response: we have addressed several of these issues (see updated page at

https://forgedb.cancer.gov/)

3. The new modular software and documentation are much improved. However, while

the response mentions Docker and dockerfiles are present in the GitHub repository,

there’s no instructions in the README for how to install the software using Docker and

Docker is not mentioned as a dependency. This seems like a relatively minor oversight

that could be corrected by adding a “Hosting FORGEdb with Docker” section to the

existing README instructions. Retaining the instructions that don’t use Docker is

valuable for users in the many institutional environments where Docker is not available.

Response: We thank the reviewer for this comment, and would like to clarify that

Docker is not needed to host. We are just using Docker to run an AWS-specific import

pipeline.

4. The instructions state that “Any static file server” can be used for hosting. While I’m

sure this is correct, it would be nice if the authors provided a recommendation and

some accompanying basic instructions so that researchers with minimal experience

can get something set up locally without needing to choose from the many static

servers that are available.

Response: We provide a minimal set of instructions for the Apache web server on

updated GitHub page, also below:

\#### Instructions

\##### Building and hosting the API

Each dataset is available for download at the following urls:

|Dataset|Version|URL|Notes|

|-|-|-|-|

|abc|v1.0|https://forgedb.cancer.gov/api/abc/v1.0/abc.forgedb.csv.gz|

|cadd|v1.0|https://forgedb.cancer.gov/api/cadd/v1.0/cadd.forgedb.csv.gz|

|cato|v1.0|https://forgedb.cancer.gov/api/cato/v1.0/cato.forgedb.csv.gz|

|closest_gene|v1.0|https://forgedb.cancer.gov/api/closest_gene/v1.0/closest_gene.forg

edb.csv.gz|

|eqtlgen|v1.0|https://forgedb.cancer.gov/api/eqtlgen/v1.0/eqtlgen.forgedb.csv.gz|

|forge2.blueprint|v1.0|https://forgedb.cancer.gov/api/forge2.blueprint/v1.0/forge2.bluepr

int.forgedb.csv.gz|

|forge2.encode|v1.0|https://forgedb.cancer.gov/api/forge2.encode/v1.0/forge2.encode.f

orgedb.csv.gz|

|forge2.erc|v1.0|https://forgedb.cancer.gov/api/forge2.erc/v1.0/forge2.erc.forgedb.csv.g

z|

|forge2.erc2-chromatin15state-all|v1.0|https://forgedb.cancer.gov/api/forge2.erc2-

chromatin15state-all/v1.0/forge2.erc2-chromatin15state-all.{0-9}.forgedb.csv.gz|{0-9}

indicates this dataset is split into 10 parts

|forge2.erc2-DHS|v1.0|https://forgedb.cancer.gov/api/forge2.erc2-

DHS/v1.0/forge2.erc2-DHS.forgedb.csv.gz|

|forge2.erc2-H3-all|v1.0|https://forgedb.cancer.gov/api/forge2.erc2-H3-

all/v1.0/forge2.erc2-H3-all.{0-9}.forgedb.csv.gz|{0-9} indicates this dataset is split into

10 parts

|forge2.forge2tf|v1.0|https://forgedb.cancer.gov/api/forge2tf/v1.0/forge2tf.forgedb.csv.g

z|

|gtex|v1.0|https://forgedb.cancer.gov/api/gtex/v1.0/gtex.forgedb.csv.gz|

|zoonomia|v1.0|https://forgedb.cancer.gov/api/zoonomia/v1.0/zoonomia.forgedb.csv.gz

|

1. `git clone https://github.com/CBIIT/nci-webtools-dceg-forgedb.git`

2. On your static file server, create an `api` folder under which you wish to host the

FORGEdb api.

3.

Create an `api` folder under which you wish to host each dataset

3.

2. Download source datasets from the table above. Create an `api`

3. Navigate to the `database` folder and run `npm install`

4. Generate a list of rsids to import as a text file, with one rsid per line

5. Run the `import.js` script. For example: `node import.js --sources sources.json --

input $PATH_TO_DATABASES_FOLDER --output $PATH_TO_OUTPUT_FOLDER --

rsids $PATH_TO_RSIDS_FILE`. Note that you can specify arbitrary datasets in

sources.json (for example, if you are creating modular builds with custom datasets).

6. Upload your output folder to any static file server, ensuring that the files are available

under the `/api` path. For example, the following url should work:

`https://your_hostname/api/${DATASET}/${VERSION}/schema.json`

\##### Building and hosting the API Client (Website)

1. `git clone https://github.com/CBIIT/nci-webtools-dceg-forgedb.git`

2. Navigate to the `client` folder and run `npm install && npm run build` to generate the

`out` folder. If your api is served under a subpath (eg:

`https://your_hostname/your_subpath/api/`), specify the subpath as the

`NEXT_PUBLIC_BASE_PATH` environment variable before building (eg: `export

NEXT_PUBLIC_BASE_PATH=/your_subpath`).

3. Upload the `out` folder to your static file server, ensuring that you do not overwrite

the contents of the `api` folder.

However, for extremely high-volume sites, we would recommend using S3/Cloudfront.

Some basic instructions for using S3/Cloudfront can be accessed at

https://docs.aws.amazon.com/AmazonCloudFront/latest/DeveloperGuide/GettingStarte

d.SimpleDistribution.html.
